# De novo and rare inherited copy-number variations in the hemiplegic form of cerebral palsy

**DOI:** 10.1038/gim.2017.83

**Published:** 2017-08-03

**Authors:** Mehdi Zarrei, Darcy L Fehlings, Karizma Mawjee, Lauren Switzer, Bhooma Thiruvahindrapuram, Susan Walker, Daniele Merico, Guillermo Casallo, Mohammed Uddin, Jeffrey R MacDonald, Matthew J Gazzellone, Edward J Higginbotham, Craig Campbell, Gabrielle deVeber, Pam Frid, Jan Willem Gorter, Carolyn Hunt, Anne Kawamura, Marie Kim, Anna McCormick, Ronit Mesterman, Dawa Samdup, Christian R Marshall, Dimitri J Stavropoulos, Richard F Wintle, Stephen W Scherer

**Affiliations:** 1 The Centre for Applied Genomics and Program in Genetics and Genome Biology, The Hospital for Sick Children, Toronto, Ontario, Canada; 2 Holland Bloorview Kids Rehabilitation Hospital, Department of Paediatrics, University of Toronto, Toronto, Ontario, Canada; 3 Deep Genomics Inc., Toronto, Ontario, Canada; 4 Department of Pediatrics, Schulich School of Medicine, Western University, London, Ontario, Canada; 5 Division of Neurology, Department of Pediatrics, Hospital for Sick Children, University of Toronto, Toronto, Ontario, Canada; 6 Thames Valley Children’s Centre, London, Ontario, Canada; 7 McMaster University, Hamilton, Ontario, Canada; 8 Grandview Children’s Centre, Oshawa, Ontario, Canada; 9 ErinoakKids Centre for Treatment and Development, Mississauga, Ontario, Canada; 10 Ottawa Children’s Treatment Centre, Ottawa, Ontario, Canada; 11 Hotel Dieu Hospital, Kingston, Ontario, Canada; 12 Department of Pediatric Laboratory Medicine, Genome Diagnostics, The Hospital for Sick Children, Toronto, Ontario, Canada; 13 Department of Molecular Genetics and McLaughlin Centre, University of Toronto, Toronto, Ontario, Canada

**Keywords:** copy-number variation, exome sequencing, hemiplegic cerebral palsy, microarray

## Abstract

**Purpose:**

Hemiplegia is a subtype of cerebral palsy (CP) in which one side of the body is affected. Our earlier study of unselected children with CP demonstrated de novo and clinically relevant rare inherited genomic copy-number variations (CNVs) in 9.6% of participants. Here, we examined the prevalence and types of CNVs specifically in hemiplegic CP.

**Methods:**

We genotyped 97 unrelated probands with hemiplegic CP and their parents. We compared their CNVs to those of 10,851 population controls, in order to identify rare CNVs (<0.1% frequency) that might be relevant to CP. We also sequenced exomes of “CNV-positive” trios.

**Results:**

We detected de novo CNVs and/or sex chromosome abnormalities in 7/97 (7.2%) of probands, impacting important developmental genes such as *GRIK2*, *LAMA1*, *DMD*, *PTPRM,* and *DIP2C*. In 18/97 individuals (18.6%), rare inherited CNVs were found, affecting loci associated with known genomic disorders (17p12, 22q11.21) or involving genes linked to neurodevelopmental disorders.

**Conclusion:**

We found an increased rate of de novo CNVs in the hemiplegic CP subtype (7.2%) compared to controls (1%). This result is similar to that for an unselected CP group. Combined with rare inherited CNVs, the genomic data impacts the understanding of the potential etiology of hemiplegic CP in 23/97 (23.7%) of participants.

## Introduction

Cerebral palsy (CP) is a permanent and nonprogressive condition that impacts posture and movement, affecting 2.0–3.5 per 1,000 live births.^1^ CP is a complex syndrome that can be defined and classified according to functional ability, topographical involvement of extremities, and neurologic patterns (spasticity, dyskinesia, and ataxia).^1^ The common neurological topography within the spastic subtype includes hemiplegia, quadriplegia, and diplegia.^1^ The Gross Motor Function Classification System (GMFCS) assesses functional motor ability, with levels from I (walks without limitations) to V (transported in a wheelchair).^2^

Hemiplegic CP is a subtype in which one side of the body is involved.^3^ It affects about 1 in 1,300 live births.^3^ This CP subtype accounted for 37.4% of CP cases in a Canadian cohort previously studied by our team.^4^ The etiology of hemiplegic CP is most commonly related to a unilateral vascular insult often involving the middle cerebral artery, a periventricular venous infarction, or a unilateral brain malformation (e.g., schizencephaly).^5^ When the injury is vascular in origin porencephaly can also be identified on brain imaging. Motor function is impaired in the body contralateral to the brain injury/anomaly. Individuals with hemiplegic CP generally are able to ambulate and function at a GMFCS level of I or II.

Along with movement and posture anomalies, CP can involve comorbidities, including seizure disorders, speech and language deficit, autism spectrum disorder (ASD), intellectual disability (ID), visual impairment, and hearing problems.^1^ Multiple risk factors are associated with CP, including the hemiplegic subtype. For example, neonatal asphyxia is thought to be a cause of related brain damage accounting for approximately 10–20% of cases.^1^

As with other neurodevelopmental disorders, genes have increasingly been found to have a role in the etiology of CP. However, the genetic findings in CP are heterogeneous, making it difficult to use them diagnostically.^4^ Twin and family studies have found that variations in genes encoding the AP-4 complex (*AP4M1*, *AP4E1*—also with an exonic deletion, *AP4B1*, and *AP4S1*)^6, 7^ and *KANK1* are associated with CP.^8^ Large chromosomal anomalies were detected in 8% of individuals in a cohort of 100 Japanese CP cases, tested using karyotyping.^9^ In a cohort of 50 cases from the South Australia CP registry, genome-wide copy-number variation (CNV) studies showed that CP was associated with rare inherited CNVs.^10^ Both rare and de novo CNVs were found associated with CP in 52 cases from Israel.^8^ A small study of 71 Japanese individuals with porencephaly or schizencephaly screened specifically for *COL4A1* variations and identified a variation in 15 (21%).^11^ Five of these individuals had a diagnosis of hemiplegic CP. Our previous study of 147 Canadian cases with parents, comprising different CP subtypes (including 37.4% with hemiplegia) revealed de novo CNVs in 7% of cases.^4^ We also found multiple large chromosomal anomalies and rare inherited variants in CP cases.^4, 12^

In the present study our objective was to assess the role of CNVs and chromosomal abnormalities in a clinically well-defined collection of individuals with hemiplegic CP to further refine the understanding of the genomic risk contribution in this common subtype of CP.

## Materials and methods

### Participants

Ninety-seven children diagnosed with hemiplegic CP (aged 2–18 years, mean=9.35, SD=4.9) were recruited and studied here for the first time. This cross-sectional study was conducted through the Childhood Hemiplegic Cerebral Palsy Integrated Neuroscience Discovery Network (CP Hemi-NET) spanning nine clinical centers across Ontario, Canada. Research Ethics Board approval was obtained from each center and all caregivers and participants (if able) provided written informed consent. Saliva samples were collected from each child and birth parents.

### Measures

#### Clinical risk factors

A retrospective health record review and a standardized parent interview identified pre-conception, pregnancy, and perinatal–neonatal CP risk factors. Examples of potential risk factors included prematurity, advanced maternal age, *in vitro* fertilization, congenital malformations, consanguinity, and a family history of CP or early stroke or heart attack at younger than 55 years. Markers of childbirth complications were also identified and pooled and included emergency C-section, Apgar score <6, resuscitation at birth, umbilical cord blood pH <7.35, seizures in the first 24 to 72 h of life, or neonatal encephalopathy.

#### Clinical outcomes

An occupational therapist evaluated the side of the hemiplegia and unilateral hand skills in the child’s hemiplegic hand using the Quality of Upper Extremity Skills Test (QUEST).^13^ QUEST scores range from 0 (no ability to use hand) to 100 (typical hand skills). GMFCS levels were obtained from the health record. Clinically ordered neuroimaging exams were reviewed for brain injury/anomaly classification. The neurodevelopmental profiles were evaluated using previously completed clinical psychoeducational assessments including IQ scores where available. Clinical diagnoses such as attention deficit/hyperactivity disorder (ADHD), ASD, and learning and intellectual disabilities were noted from the psychology or health record. A summary of the CP risk factors and clinical outcomes for a selected number of CNV-positive cases is found in Supplementary Table S1 online.

### Genotyping and variant calling

Genomic DNA extracted from saliva was genotyped on the Affymetrix CytoScan HD platform at The Centre for Applied Genomics.^4^ Relevant microarray data are deposited in the Gene Expression Omnibus (https://www.ncbi.nlm.nih.gov/geo/; GSE80813). The quality control and ancestry assessment procedures were as discussed previously.^4^ The ancestry of samples was determined using PLINK v1.90b2, finding 67 families of European ancestry, and 30 from other backgrounds (Supplementary Table S2).

We called CNVs as previously described (Supplementary Information). The genomic coordinates used are based on Human Genome Build GRCh37/hg19.

### Detection of rare variants

We identified rare CNVs as those at not more than 0.1% frequency among 10,851 population control samples (Supplementary Figure S1), using a 50% reciprocal overlap strategy.^14^ We further restricted our list to those with more than 75% overlap with copy-number stable regions, according to our stringent CNV map of the human genome (see Supplementary Information).^15^ CNVs deemed relevant, as indicated below, were validated using an SYBR Green-based real-time quantitative PCR assay, TaqMan Copy Number Assays, or droplet digital PCR (ddPCR) (Supplementary Table S3).

### Brain-expressed critical exons and burden of pLI scores

We tested the burden of critical exons as identified by Uddin et al.^16^ in genes whose coding sequences were impacted by rare CNVs, in cases compared with controls, for 16 different brain regions (see Supplementary Information). We performed our analyses at three developmental time points: prenatal (12–37 weeks of gestation), early postnatal and adolescence (4 months–15 years) and adult (>18 years).

We also obtained the probability of truncating loss-of-function intolerance (pLI)^17^ for genes impacted by deletions in our cases compared with all 10,851 control subjects at the individual level. We compared cases to controls using an unpaired *t*-test between sums of pLI scores in cases compared with controls to evaluate significance in genetic burden.

### Exome sequencing

We undertook exome sequencing to search for potential smaller sequence-level variants not found by microarrays in the 23 families with CNV findings most relevant to CP (Table 1). Our rationale was to investigate whether additional mutations identified might help explain the hemiplegic CP itself or some other medical comorbidity. Exome sequencing was conducted using the Illumina HiSeq2500 at the Centre for Applied Genomics. Sequencing data analysis, alignment, variant calling, and filtering were as described previously.^18^ We investigated damaging variants and those impacting genes previously implicated in neurodevelopmental or neuromuscular disorders.

## Results

Our hemiplegic cohort had a sex ratio of 1.5 (59 male: 38 female subjects), which is slightly higher than that reported from the general CP population (1.3).^19^ There was a balanced distribution of left (52.6%) and right (47.3%) hemiplegia. The mean QUEST score was 64 (SD=29.5) and GMFCS levels ranged from I to III with a median level of I.

We detected 340 stringent CNVs in proband DNA (Supplementary Table S2). These ranged from 10 kb to a duplication of the entire X chromosome. The majority of CNVs were deletions (n=222; 65%) and 118 were duplications (35%). Of these, 129 (38%) affect coding sequences of genes, and 62% involved noncoding regions of the genome.

### Clinically relevant variants

In 23 individuals, 30 CNVs with potential relevance to CP were identified (Table 1). These CNVs were either de novo (nine events in seven cases), or involved regions associated with known DECIPHER syndrome loci or ClinGen loci (three cases; one de novo and two paternally inherited), impacted a gene with an established disease role in the brain or muscle, or involved in ASD or comorbid features of CP such as seizure or hearing loss (19 events in 16 cases).

**Figure 1:**
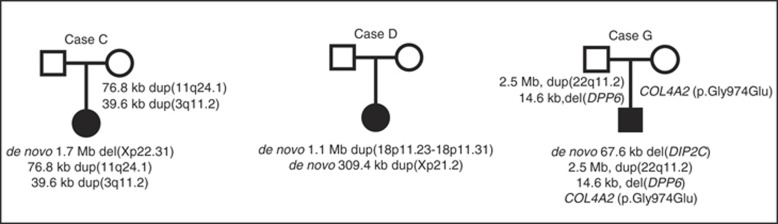
Pedigrees of selected families (cases C, D, and G) with multiple variants.

We identified de novo CNVs in 7/97 (7.2%) patients. These included a CNV congruent with the steroid sulfatase deficiency DECIPHER syndrome locus, and one instance of Klinefelter syndrome (XXY chromosomes; Table 1). We identified two patients each with two different de novo CNVs. Case B had a 70.4 Mb duplication encompassing the whole p arm and part of the q arm of chromosome X, and a deletion of the rest (84.9 Mb) of the q arm (Turner syndrome). This 70.4 Mb duplication was confirmed to be mosaic by ddPCR. The other, Case D, carried a duplication in 18p11.23–18p11.31, impacting three genes—*LAMA1, PTPRM*, and *LRRC30*—and a duplication in Xp21.2 impacting the *DMD* gene (Figure 1). We also found a 67.6 kb mosaic deletion impacting at least two exons of the *DIP2C* gene in two unrelated cases (cases F and G). In addition, we observed a de novo deletion impacting the same segment (at least two exons) of this gene in a case first reported in our previous publication.^4^ We validated this deletion and refined its boundaries in those three cases using eleven different TaqMan assays by ddPCR (see Supplementary Information for experiments performed on these three cases). Genotype data suggest this deletion may be somatic in origin, necessitating testing in additional tissues for confirmation.

Of 21 rare inherited CNVs, 14 were inherited maternally and seven paternally. Two of the latter involved DECIPHER syndrome loci: 22q11.2 duplication syndrome (case G; Figure 1) and hereditary neuropathy with liability to pressure palsies syndrome (case H). Of the remaining inherited CNVs, 12 were duplications and 7 were deletions.

### Critical exons and pLI scores

**Figure 2:**
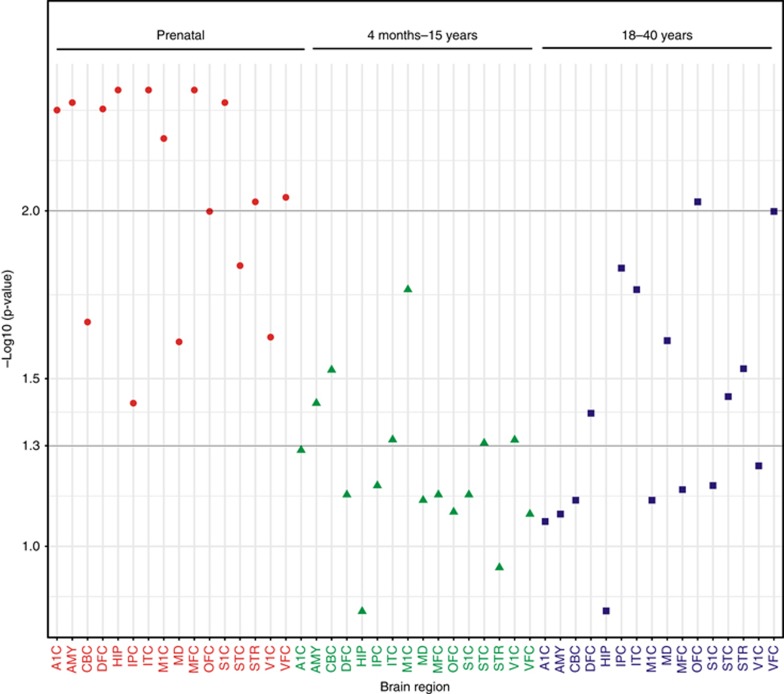
**Critical exons in genes expressed prenatally are significantly impacted by rare CNVs in 16 different brain regions.** The *P* value is corrected using the Benjamini-Hochberg method. AMY, amygdaloid complex; CBC, cerebellar cortex; V1C, primary visual cortex; STC, posterior (caudal) superior temporal cortex; IPC, posterior inferior parietal cortex; A1C, primary auditory cortex; S1C, primary somatosensory cortex; M1C, primary motor cortex; STR, striatum; DFC, dorsolateral prefrontal cortex; MFC, medial prefrontal cortex; VFC, ventrolateral prefrontal cortex; OFC, orbital frontal cortex; MD, mediodorsal nucleus of thalamus; ITC, inferolateral temporal cortex; HIP, hippocampus.

We observed an enrichment of brain critical exons among CNVs in cases compared with the platform-matched controls (see Supplementary Information). The difference was significant (*P*<0.05) for exons with prenatal expression in 16 brain regions, whereas only amygdaloid complex, cerebellar cortex, and primary motor cortex showed such a difference in the exons with expression from childhood to adolescence (Figure 2). CNVs in CP cases were enriched for exons expressed in adults in these regions.

Mean pLI scores were higher, but not statistically significant, in our cases compared with controls genotyped on platforms other than CytoScan HD (0.267 vs. 0.096; P=0.109) and with platform-matched controls (0.267 vs. 0.10; P=0.118).

### Exome sequencing

We found 30 de novo variants from 17 cases (Supplementary Table S4). We identified potential risk variants in two cases. Case E has a nonsynonymous de novo single nucleotide variant (SNV) in *ENTPD1,* which is recessive and involved in spastic paraplegia (OMIM 615683). This case also carries a nonsynonymous de novo SNV in *NOVA1*, a Nova-family RNA-binding protein, important for splicing regulation in neurons, predicted to be haploinsufficient (pLI=0.98), and resulting in motor dysfunction when knocked out in mouse (OMIM 602157). The second case, Case L, has a nonsynonymous de novo SNV in *ATAD3C*, also involved in various neurological disorders.^20^ We also found 45 rare inherited SNVs impacting the neurodevelopmental or neuromuscular disorders from 21 cases. We additionally identified two different damaging missense variants in *COL4A2*, which is involved in muscular disorders (OMIM 120090), inherited from the mother of case G (Figure 1) and from the father of case Q.

### Stratifying cases into two groups

We stratified cases into two categories: those with identified CNV risk (n=23) and those without a CNV risk (n=74) (Table 2). We found that there were significantly more females in the CNV group (*P*=0.006). Among potential risk factors for CP, there was a significant difference in gestational term between the groups (*P*=0.02), with the CNV group having a lower rate of prematurity. Levels of factors such as advanced maternal age, use of *in vitro* fertilization, the presence of congenital malformations, and markers of childbirth complications, among others, were not found to be significantly different between the two groups.

### Notable case findings

#### De novo CNVs and CNVs known to be associated with DECIPHER syndrome loci

**Case A** has sex chromosome aneuploidy with a diagnosis of Klinefelter syndrome (47, XXY). Neurological disorders such as CP, ADHD, epilepsy, and delayed motor milestones have been reported in some patients with Klinefelter syndrome.^21^

**Case B** carries a complex rearrangement: a mosaic duplication (70 Mb) spanning chromosome Xp and the centromere side of Xq. She also has an 84.9 Mb deletion of distal Xq starting from the same breakpoint as where the duplication ends (Table 1; Supplementary Figure S2). The retained copy of the q arm is maternal in origin. This might reflect an iso-dicentric chromosome X with a terminal deletion of paternal origin, but confirmation by karyotype is needed. Delays in gross motor milestones and reduced general muscle tone and strength are reported in cases with X-chromosome monosomy.^21^ Since both deletions and duplications of chromosome X are associated with delayed motor functions, we consider whether CP in this patient may be explained by these chromosomal anomalies. Her intellectual ability was unknown. Her mother’s maternal age was advanced.

**Case C** carries one de novo and two maternally inherited CNVs (Figure 1). The de novo variant is a 1.7 Mb deletion on Xp, impacting five genes including *STS*, which is associated with steroid sulfatase deficiency (Supplementary Figure S3). Others studied a link between haploinsufficiency of *STS* and neurodevelopmental disorders, including intellectual disability and autism in a girl.^22^ Her inherited variants include a 39.6 kb duplication impacting *EPHA6*, a gene implicated in autism (Supplementary Figure S4) and a 76.8 kb duplication impacting *SCN3B* (Supplementary Figure S5). The latter gene encodes a member of the sodium channel subunit in neurons and muscle cells; mutations in such genes are involved in epilepsy,^23^ a comorbidity of CP. This case has a family history of CP and early stroke.

**Case D** has a language-based learning disability, is functioning at a GMFCS level of I, and carries two de novo duplications (Table 1 and Supplementary Table S1; Figure 1). The first CNV is a 309 kb duplication impacting the first 15 exons of the *DMD* gene (all exons for five isoforms), and a single exon of the *FTHL17* gene (Supplementary Figure S6). Duplication of *DMD* was reported in a female with spastic right hemiplegia.^4^ The second CNV is a 1.15 Mb duplication of 18p11.23–18p11.31, impacting three genes: *LAMA1*, *PTPRM*, and *LRRC30* (Supplementary Figure S7). Protein-truncating variants in the *LAMA1* gene have been linked to congenital muscular dystrophy, myopia, and retinal dystrophy.^24^

**Case E**, who has normal intellectual development, harbors a 475.1 kb de novo CNV deletion impacting *GRIK2* (Supplementary Figure S8 and Supplementary Table S1), which belongs to the kainate family of glutamate receptors. Haploinsufficiency of members of this family can cause intellectual disability.^25^ She also carries a de novo damaging point mutation in *NOVA1*, which may contribute to her phenotype (OMIM 615683).

**Cases F** and **G** each have a de novo 67.6 kb deletion impacting at least two exons in the *DIP2C* gene (Figure 1). Case F has ADHD (inattentive subtype), is a slow learner, and has a proximal middle cerebral artery infarction. Case G has a family history of CP (mother and maternal second cousin) and he had seizures on his third day of life. No imaging was available for this case. Genetic variants of *DIP2A*, *DIP2B,* and *DIP2C* have a possible role in developmental dyslexia, intellectual disability, and developmental delay.^26^ Among ASD cases one inherited frameshift and two de novo mutations in the *DIP2C* gene have been reported.^27, 28^ Case G also carries two paternally inherited CNVs and a point mutation in *COL4A2*, but none in *DIP2C* (Figure 1). One CNV is a 2.5 Mb duplication reciprocal to the 22q11.2 deletion syndrome locus, which is sometimes associated with ASD.^14^ This patient also carries a 14.6 kb deletion impacting the *DPP6* gene (Supplementary Figure S9). This gene has been associated with a number of human central nervous system disorders, including ASD, intellectual disability, and spinal muscular atrophy.^29^ This patient also carries a heterozygous point mutation inherited from his mother in the *COL4A2* gene (c.2921G>A; p.Gly974Glu; identified by gene-panel testing by clinical geneticists at the Hospital for Sick Children; and confirmed for this study with exome and Sanger sequencing). Mutations in this gene, which is expressed in the basement membrane during early development, have been reported in patients with muscular cramps, cerebral small-vessel disease, and hemorrhagic stroke (OMIM 120090).

**Case H** is functioning at a GMFCS level I and carries a 1.4 Mb paternally inherited deletion in 17p12, a region associated with hereditary neuropathy with liability to pressure palsies syndrome, characterized by muscle weakness (OMIM 162500).

#### Inherited CNVs impacting neurodevelopmental or muscular risk genes

In addition to two CNVs associated with DECIPHER syndrome loci discussed above (Cases G and H), we observed 19 inherited CNVs from 18 patients (18/97=18.6%). We describe three of these here, and the rest in the Supplementary Information.

**Case I,** who has a normal intellectual profile, carries a 1.03 Mb paternally inherited deletion impacting the first exon of the *CNTN4* gene and all except the first two exons of the *CNTN6* gene (Supplementary Figure S10). These two genes belong to the family of contactin genes (*CNTN*s) with a role in formation of axon connections in the developing nervous system. Deletions of *CNTN*s have been linked to hypotonia, psychomotor retardation, ASD, intellectual disability, developmental delay, microcephaly, and other neurodevelopmental disorders.^30^

**Case L** has a language-based learning disability. She carries a maternally inherited 370.6 kb duplication impacting *ABAT*, *USP7*, and four other genes (Supplementary Figure S11). De novo CNVs, including duplications, impacting the *USP7* gene, are implicated in ASD and schizophrenia.^31^ The *ABAT* deficiency phenotype includes psychomotor retardation and seizures. Single nucleotide variants in the *ABAT* gene, probably disrupting its protein function, may have a link to autism.^32^

**Case Q**, who has a normal developmental profile, carries a maternally inherited 114.4 kb deletion impacting exons 8 and 9 of the *CNTNAP2* gene (Supplementary Figure S12), which encodes a member of the neurexin family with function in the nervous system. Deletions, including only a single exon of this gene, have been implicated in dyslexia, apraxia, language impairment, motor regression, and intellectual disability.^33^ This case also carries a paternally inherited potentially damaging missense SNV in *COL4A2*.

## Discussion

We found de novo CNVs in 7.2% of cases in this well-defined collection of individuals with hemiplegic CP. This was comparable to the 7% we previously reported in a cohort of individuals with CP from all subtypes,^4^ but higher than the de novo CNV rate in other neurodevelopmental disorders, including idiopathic ASD, ADHD, ID, and schizophrenia (reviewed by Oskoui et al.^4^). However, in a cohort of CP cases from Israel, 13% had de novo CNVs.^8^ Taken together, these findings suggest that de novo CNVs may be contributing to the etiology of various subtypes of CP, including hemiplegia.

The contribution of rare inherited CNVs to the etiology of hemiplegia has been reported previously, but only in mixed cohorts, rather than a purely hemiplegic cohort, as reported here. Our group identified two inherited duplications in cases with spastic right hemiplegia,^4^ and McMichael et al.^10^ identified rare inherited CNVs in cases diagnosed with hemiplegia. Here, in 18.6% (18/97) of our subjects, we found rare inherited CNVs (63% duplications), impacting genes expressed in the brain or congruent with genomic disorders. For example, brain-expressed genes in the neurexin or contactin, and immunoglobulin gene families, (e.g., *CNTNAP2*, *CNTN4*, *CNTN6)*, or genes encoding members of potassium or sodium channels (i.e., *KCNK9*, *KCNJ9*, *KCNJ10*, and *SCN3B*), were perturbed in our hemiplegic CP cohort. Alterations of these genes are risk factors for other neurodevelopmental disorders. For inherited variants, many factors may explain the phenotype discordance between child and parent, including variable expressivity, incomplete penetrance, and epigenetic modification of gene expression. Of those genes impacted by the de novo and inherited CNVs in this study, *GRIK2*, *KCNJ9*, *KCNJ10*, *GLGAP1*, *PTPRM*, and *DPP6* are clustered together in a protein interaction network (Supplementary Figure S13).

Inflammation, including chorioamnionitis and congenital infections, is an established risk factor for all CP subtypes including hemiplegia, owing possibly to abnormalities in the inflammatory response system.^34^ An earlier study established a link between a SNP (rs1800795) in interleukin-6 (*IL-6*) and CP.^34^ However, we found no CNVs impacting genes involved in the inflammatory response, including those highlighted in two population-based studies.^35, 36^

The enrichment of brain-expressed critical exons impacted by CNVs in our cases (as seen in other neurodevelopmental disorders such as ASD^16^) may explain factors contributing to the etiology of hemiplegia as well as the associated neurodevelopmental comorbidities. The primary motor cortex and cerebral cortex may be particularly relevant to core phenotypes of the hemiplegic CP. Although the scores for the pLI burden in our cases were not significantly elevated, the higher mean score may provide additional evidence that perturbed genes would be a contributory factor in the etiology of CP.

Among individuals with CP including hemiplegia, about 20% have neurodevelopmental disorders and 9% have ASD.^37^ Multiple de novo CNVs impacting genes implicated in ASD were found in our CP cases. For example, cases E, F, and G have de novo deletions impacting exons of *GRIK2* and *DIP2C,* with a pLI score corresponding to be highly constrained (pLI>0.9; Table 1 and **Supplementary Table S5**). We also found rare inherited exonic deletions and duplications impacting other ASD risk genes, e.g., *KCNK9*, *CNTN6*, *CNTN4*, *ASTN2*, and *MCPH1*, in our CP cases (Table 1). In our CNV-positive cases, only case P, with a 22q11.21 duplication, has ASD (as well as ADHD). We also uncovered CNVs at three genomic disorder loci: a *de novo* deletion of Xp22.31associated with steroid sulfatase deficiency, a 22q11.21 duplication, and a 17p12 deletion (both inherited).

In our sample we found that the CNV group had proportionally more females and lower rates of prematurity than the non-CNV group. Clinically, CP cases are more common in males than females (with a ratio of 1.3, or 1.5 in the current study) although the exact risk mechanism has not been well established. However, as with autism,^14^ females were represented more frequently in the CNV-positive cases (*P*=0.006). Prematurity is also a significant CP risk factor, increasing the chance of developing CP 100-fold.^38^ However, in this study, term gestation was more common in the CNV-positive cases. The increased prevalence of females and children born at term in the CNV-positive group could be explained by the presence of a CNV itself being a risk factor that contributes to the etiology of hemiplegic CP. This could also explain the overrepresentation of other risk factors, such as higher rates of males and prematurity (*P*=0.02), in the non-CNV group (Table 2).

We identified potentially damaging point mutations in *COL4A2*, a gene associated with hemorrhagic stroke, in cases G and Q (OMIM 120090), thus contributing to the understanding of stroke in the etiology of hemiplegic CP. Birth asphyxia (neonatal encephalopathy) has been associated with CP in 10%–20% of individuals.^1^ Genetic variations could also underlie vulnerabilities that lead to birth asphyxia.^39^ The presence of potentially harmful CNVs in 23.7% (23/97) of our cohort may provide further evidence for this hypothesis, although we did not identify a difference in childbirth complications in the CNV, compared to the non-CNV, group.^38^ However, the CP risk factors of advanced maternal age, use of *in vitro* fertilization, and the presence of congenital malformations were not significantly different between these two groups.

Our results show that hemiplegic CP can be associated with de novo or rare inherited CNVs. The presence of de novo CNVs and those involving well-defined genomic disorders among our cohort suggests the benefit of genomic testing for diagnostic purposes in hemiplegic CP. DOPA-responsive dystonia and hereditary spastic paraplegia (HSP) can present in asymmetric neurologic presentations with one side more involved in the early stages, and are conditions with identified genetic mutations in the *GCH1* and *ATL1* genes, respectively, but may be misdiagnosed as CP.^40^ The distinction is important, as DOPA-responsive dystonia can be treated with medications such as levodopa, and the clinical trajectory of HSP is different from that of CP as the neurologic symptoms progress. Therefore, genetic testing, particularly whole-genome sequencing, which has the potential for detecting CNVs and smaller variants, should be considered as part of the diagnostic assessment of an individual presenting with CP symptoms, for ruling out neurologic disorders with similar phenotypes, or garnering a better understanding of the etiology of CP for that particular individual.

## Figures and Tables

**Table 1:** Copy-number variations of potential significance in 23 CP subjects

**Case**^a^	**ID**	**Sex**	**Type**^b^	**Location**	**Size (bp)**^c^	**Genes**^d^	**Inheritance**	**CNV pathogenicity**	**Neurodevelopmental profile**^e^
De novo									
Case A^f^	200117	M	dup	Xp22.33–Xq28	155,270,560	826 genes	De novo	Pathogenic^g^	Normal intellectual development
Case B^h^	234968	F	del	Xq13.1–Xq28	84,886,227	457 genes	De novo	Pathogenic^i^	N/A
			dup^j^	Xp22.33–Xq13.1	70,384,332	369 genes	De novo	Pathogenic	
Case C^f^	212306	F	del	Xp22.31	1,686,421	*STS* + 4 genes	De novo	VOUS^k^	Normal intellectual development
			dup	11q24.1	76,847	*SCN3B*, *GRAMD1B*	Maternal	VOUS	
			dup	3q11.2	39,636	*EPHA6*	Maternal	VOUS	
Case D^h^	208937	F	dup	18p11.23, 18p11.31	1,146,375	*LAMA1, PTPRM, LRRC30*	De novo	VOUS	Language-based learning disability
			dup	Xp21.2	309,376	*DMD*, *FTHL17*	De novo	VOUS^l^	
Case E^f^	216072	F	del	6q16.3	475,077	*GRIK2*	De novo	VOUS^m^	Normal intellectual development
Case F^f^	216185	M	del^j^	10p15.3	67,632	*DIP2C*	De novo	VOUS	ADHD-Inattentive sub-type, slow learner
Case G^h^	222710	M	del^j^	10p15.3	67,632	*DIP2C*	De novo	VOUS	Normal learning profile
			del	7q36.2	14,653	*DPP6*	Paternal	VOUS	
*Inherited: DECIPHER syndromes /cytogenetic chromosomal anomalies*									
Case G^h^	222710	M	dup	22q11.21	2,548,820	45 genes	Paternal	Likely pathogenic^n^	Normal learning profile
Case H^h^	216192	M	del	17p12	1,395,494	*PMP22* + 8 genes	Paternal	Pathogenic^o^	N/A
*Inherited: CNVs impacting neurodevelopmental or muscular function genes*									
Case I^h^	219594	M	del	3p26.3	1,027,287	*CNTN6, CNTN4, CNTN4-AS2*	Paternal	VOUS	Normal learning profile
Case J^f^	198195	M	dup	8p23.1, 8p23.2	973,229	*MCPH1* + 10 genes	Maternal	VOUS	Normal intellectual development
Case K^h^	214281	F	dup	5q11.2	504,651	*HSPB3*, *ARL15*	Maternal	VOUS	Normal learning profile
Case L^f^	216197	F	dup	16p13.2	370,593	*ABAT*, *USP7* + 4 genes	Maternal	VOUS	Language-based learning disability
Case M^f^	217737	M	dup	8q24.3	255,856	*KCNK9*	Maternal	VOUS^p^	Language-based learning disability
			dup	7q36.3	251,267	*NCAPG2*, *ESYT2*	Maternal	VOUS	
Case N^f^	247947	F	dup	1q23.2	196,398	*KCNJ10*, *KCNJ9* + 5 genes	Maternal	VOUS	Normal intellectual development
Case O^f^	217932	M	del	3q26.31	176,370	*NAALADL2*	Maternal	VOUS	N/A
Case P^f^	208289	F	dup	22q11.21	162,405	*DGCR2*, *DGCR14* + 4 genes	Maternal	VOUS	ASD, ADHD
Case Q^f^	221713	F	del	7q35	114,399	*CNTNAP2*	Maternal	VOUS	Normal learning profile
Case R^f^	208341	F	del	5p15.31	77,527	*SEMA5A*	Maternal	VOUS	Language-based learning disability
Case S^f^	234267	F	dup	9q33.1	40,814	*ASTN2*	Paternal	VOUS^q^	Normal intellectual development
Case T^h^	239838	F	del	22q12.3	34,122	*RFPL2*, *SLC5A4*	Paternal	VOUS	Normal intellectual development
Case U^h^	208290	F	del	18p11.31	25,582	*DLGAP1*	Maternal	VOUS	Normal learning profile
Case V^h^	199743	F	dup	16q24.3	24,064	*ZNF778*	Maternal	VOUS	Intellectual disability
Case W^f^	209038	F	dup	17p11.2	16,602	*MYO15A*	Paternal	VOUS^r^	Normal intellectual development

CNV, copy-number variant; CP, cerebral palsy; del, deletion; dup, duplication; F, female; M, male; VOUS, variant of uncertain significance with respect to CP and comorbid features.

a Rare CNVs for all individuals are provided in Supplementary Table S2.

b Each CNV validated by SYBR Green–based real-time quantitative PCR, or TaqMan assay, or droplet digital PCR (ddPCR) with TaqMan assay (see Supplementary Table S3).

c The actual coordinates are in Supplementary Table S2.

d The probabilities of truncating loss-of-function intolerance (pLI) for genes impacted by CNVs are shown in Supplementary Table S5.

e Refer to Supplementary Table S1 for detailed clinical information.

f Right hemiplegia CP.

g Klinefelter syndrome (47,XXY).

h Left hemiplegia CP.

i Turner syndrome.

j Mosaic variants validated using ddPCR with TaqMan assay (see Supplementary Information for additional information).

k Male carrier of this recurrent deletion is associated with ichthyosis. No phenotype in females.

l Partial duplication of *DMD* may or may not affect expression.

m *GRIK2* associated with autosomal recessive mental retardation. Deletion of one copy not expected to be pathogenic.

n 22q11.2 duplication syndrome.

o Recurrent deletion is associated with hereditary neuropathy with liability to pressure palsies syndrome.

p Partial duplication of *KCNK9* may or may not affect expression of this gene.

q Intragenic duplication may disrupt protein.

r *MYO15A* is associated with autosomal recessive deafness.

**Table 2:** Characteristics of patient groups with and without CNVs

	CNV^a^	Non-CNV^a^	Test statistic	Significance
*Participant characteristics*				
Age (years) χ (s)	8.35 (4.84)	9.67 (4.90)	−1.1^b^	0.26
Sex				
Male	8 (34.8%)	51 (68.9%)	8.6^b^	0.006
Female	15 (65.2%)	23 (31.1%)		
Hemiplegia *n* (%)				
Left	13 (56.5%)	37 (51.4%)		
Right	10 (43.5%)	35 (48.6%), M=2	1.1^b^	0.58
Neuroimaging *n* (%)				
MCA	7 (30.4%)	24 (32.4%)		
PVI	10 (43.5%)	30 (40.5%)	2.9^b^	0.24
Other^c^	1 (4.34%)	4 (5.4%)		
Not available	5 (21.7%)	16 (21.6%)		
Quality of Upper Extremity Skills Test score χ(s)	63.24 (27.1)	63.79 (30.5)	−0.1^d^	0.94
Full Scale IQ percentile score^e^ χ(s)	35.47 (26.9)	46.08 (31.1)	−1.2^d^	0.28
*Potential risk factors for CP*				
Advanced maternal age (≥35 years) *n* (%)	9 (39.1%)	18 (25.7%), M=4	1.5^b^	0.29
Family history of genetic risk factors^f^ *n* (%)	4 (17.4%)	15 (20.8%), M=2	0.8^b^	0.68
Use of *in vitro* fertilization *n* (%)	1 (4.3%)	3 (40.5%)	1.1^b^	0.59
Gestation *n* (%)				
Prematurity (<37 weeks)	1 (4.3%)	20 (27.0%)		
Term (≥37 weeks)	21 (91.3%)	47 (63.5%)	5.9^b^	0.02
Unavailable	1 (4.3%)	7 (9.5%)		
Small or large birth weight for gestational age^g^ *n* (%)	6 (27.3%), M=1	10 (15.4%), M=9	1.6^b^	0.22
Congenital malformations *n* (%)	6 (26.1%)	14 (18.9%)	0.6^b^	0.56
Birth process complications^h^ *n* (%)	14 (60.9%)	46 (63%), M=1	0.03^b^	0.52

The sample had the following functional classification levels: Gross Motor Functional Classification level I (85.5%), II (10.5%), and III (3.9%); Manual Abilities Classification System level I (50%), II (39.7%), III (8.6%), and IV (1.7%); and Communication Function Classification System level I (75.5%), II (13.8%), III (5.2%), and IV (2.1%).

CNV, copy-number variation; M, missing data number per cell; MCA, middle cerebral artery; PVI, periventricular infarct; s, standard deviation; χ, mean.

a Numbers of individuals in the CNV and non-CNV groups are 23 and 74, respectively.

b Chi-square.

c Other: for example, non-middle cerebral artery stroke, brain malformation.

d *t*-test.

e IQ testing was assessed only in children >4 years old.

f ”Family history of genetic risk factors” was defined as participants having a1st- or 2nd-degree relative with CP, early stroke or heart attack (<60 years old), or consanguinity. Consanguinity is defined as a hereditary relationship between biological parents or grandparents of the index child that is the result of having a common parent or other ancestor.

g Small birth weight for gestational age ≤10th percentile; high birth weight for gestational age ≥97th percentile.

h ”Birth process complications” was defined as having undergone an emergency cesarean section, had an Apgar score <6 at five minutes, undergone resuscitation at birth, had umbilical cord blood pH <7.35, or seizures in the first 24 to 72 h of life, and/or neonatal encephalopathy.
