# Supplementary material for: De novo and rare inherited copy-number variations in the hemiplegic form of cerebral palsy
Source: Genet Med. 2017 Aug 3;20(2):172–80. doi: 10.1038/gim.2017.83 (PMC5846809; doi:10.1038/gim.2017.83)
Supplement: Supplementary Table S1 [file gim201783x1.pdf]

Table S1. Hemi-NET CNV cases with positive clinical risk factors

Case number or ID	Risk factors: preconception	Risk factors: pregnancy	Risk factors: perinatal-neonatal	Neuroimaging category	QUEST score	Neurodevelopmental Profile
**Case A**<br>$G_1P_1A_0$, Singleton<br>Term<br>Male<br>R hemiplegic CP<br>GMFCS I				PVI-V<br>PV-GM	64	- Normal intellectual development (based on WPPSI-III)
**Case B**<br>$G_{11}P_3A_8$, Singleton<br>Term<br>Female<br>L hemiplegic CP<br>GMFCS I	-Advanced maternal age (40yrs)<br>-A_8=8 miscarriages<br>-P_3=1 still-birth >20wks gestation<br>-Family hx of early heart attack	-IVF<br>-PV bleeding: 1st trimester	-SGA: BW5th-10th%ile	Focal porencephaly.	22	N/A
**Case C**<br>$G_2P_2A_0$, Singleton<br>Gestational age unknown<br>Female<br>R hemiplegic CP<br>GMFCS I	-Family hx of CP and early stroke	-PV bleeding: 2nd trimester	-Acidosis (pH=7.25)	PVI-V	92	Normal intellectual development (based on WPPSI-III)
**Case D**<br>$G_3P_2A_1$, Singleton<br>Term<br>Female<br>L hemiplegic CP<br>GMFCS II	-A_1=1 miscarriage<br>-Family hx of early stroke and clots	-PIH<br>-PV bleeding: 1st trimester	-Emergent C-section (non-communicating hydrocephalus; identified via bedside U/S during labour)<br>-Acidosis (pH=7.17)	Hydrocephalus	50	-Language-based learning disability
**Case E**<br>$G_3P_1A_2$, Singleton<br>Term<br>Female<br>R hemiplegic CP<br>GMFCS I	-A_2=2 miscarriages<br>-Family hx of early heart attack		-Emergent C-section (non-reassuring FHR)	PVI-V	69	Normal intellectual development (based on WPPSI-III)
**Case F**<br>$G_1P_2A_0$, Multiple: Twin A<br>Term<br>Male<br>R hemiplegic CP<br>GMFCS I		-Medication use: Amitriptyline<br>-Multiple pregnancy: twins		MCA-PM1	35	-ADHD-Inattentive sub-type<br>-Slow learner
**Case G**<br>$G_3P_2A_3$, Multiple: Triplet (C)<br>Term<br>Male<br>L hemiplegic CP<br>GMFCS I	-Advanced maternal age (35yrs)<br>-Family hx of CP (mother, maternal second cousin)<br>-A_3= 1 miscarriage<br>- variation in the COL4A2 gene identified	- Triplet pregnancy with in utero death of triplets A and B at 9wks gestation<br>-PV bleeding: 1st trimester	-Acidosis (pH=7.33)<br>-Seizures on day 3 of life	No image	39	-Normal learning profile (as identified by a developmental pediatrician)
**Case H**	-Advanced maternal age (35yrs)	-PV bleeding: 1st trimester		PVI-V	82	N/A

Table S1. Hemi-NET CNV cases with positive clinical risk factors

$G_3P_3A_0$, Singleton<br>Term<br>Male<br>L hemiplegic CP<br>GMFCS I	-Family hx of early heart attack			IVH		
**Case I**<br>$G_2P_1A_1$, Singleton<br>Term<br>Male<br>L hemiplegic CP<br>GMFCS I	-A_1=1miscarriage	-Toxin exposure: ETOH use during pregnancy	-Emergent C-section (non-reassuring FHR w/FTP)<br>-Meconium staining<br>-Acidosis (pH=7.30)	No image	100	-Normal learning profile (as identified by a developmental pediatrician)
**Case J**<br>$G_2P_2A_0$, Singleton<br>Term<br>Male<br>R hemiplegic CP<br>GMFCS I	-Advanced maternal age (35yrs)<br>-Family hx of early heart attack<br>P_2=1 still-birth > 20wks gestation		-Emergent C-section (FTP)	MCA-DM1	50	Normal intellectual development (based on WPPSI-III)
**Case K**<br>$G_2P_1A_1$, Singleton<br>Term<br>Female<br>L Hemiplegic CP<br>GMFCS I	-Advanced maternal age (37yrs)<br>-A_1= 1 miscarriage	- PV bleeding: 1st trimester<br>- Preeclampsia<br>- Chorioamnionitis	-Assisted delivery (forceps)<br>-Maternal Fever<br>-Acidosis (pH=7.3)<br>-Jaundice	PVI - V	91	-Normal learning profile (as identified by a developmental pediatrician)
**Case L**<br>$G_2P_2A_0$, Singleton<br>Term<br>Female<br>R hemiplegic CP<br>GMFCS I	-Family hx of early heart attack		-LGA: BW>97th %ile	MCA-DM1	61	-Language-based learning disability
**Case M**<br>$G_4P_2A_2$, Singleton<br>Term<br>Male<br>R hemiplegic CP<br>GMFCS I	-A2=1miscarriage	-PIH<br>-Pre-eclampsia	-Emergent C-section (non-reassuring FHR)<br>-Tight nuchal cord<br>-NEC<br>-SGA: BW<3rd%ile	No image	31	-Language-based learning disability
**Case N**<br>$G_7P_2A_5$, Singleton<br>Term<br>Female<br>R hemiplegic CP<br>GMFCS I	-Advanced maternal age (41 yrs)<br>-A_5=4 miscarriages		-SGA: BW<3rd%ile	PVI-V	97	-Normal intellectual development based on WISC-IV
**Case O**<br>$G_2P_2A_0$, Singleton<br>Term<br>Male<br>R hemiplegic CP	-Family hx of CP (paternal uncle) and early heart attack		-Emergent C-section (FTP)<br>-Cord knot<br>-Seizures on day 1 of life	PCA-PM1	9	N/A

Table S1. Hemi-NET CNV cases with positive clinical risk factors

GMFCS I						
**Case P**<br>$G_1P_1A_0$, Singleton<br>Term<br>Female<br>R Hemiplegic CP<br>GMFCS I			-Seizures on day 4 of life	MCA-PM1	38	-ADHD<br>-ASD
**Case Q**<br>$G_2P_1A_1$, Singleton<br>Term<br>Female<br>R hemiplegic CP<br>GMFCS I	-Advanced maternal age (35yrs)<br>-Family hx of early heart attack	-PV bleeding: 1st trimester		MCA-PT	98	-Normal learning profile (as identified by a developmental pediatrician)
**Case R**<br>$G_1P_1A_0$, Singleton<br>Term<br>Female<br>R hemiplegic CP<br>GMFCS I			-Assisted delivery (forceps)<br>-Abx's administered $\geq$ to 10 days during neonatal period<br>-Meconium staining<br>-Acidosis (pH=7.05)<br>-LGA: BW>97th%ile	PVI-V	97	-Language-based learning disability
**Case S**<br>$G_2P_2A_0$, Singleton<br>Term<br>Female<br>R hemiplegic CP<br>GMFCS I	-Advanced maternal age (41yrs)	-PV bleeding: 3rd trimester	-Emergent C-section (FTP)<br>-Cord prolapse during labour<br>-Nuchal cord (x2, loose)<br>-LGA: BW>97th%ile	No image	83	-Normal intellectual development (based on WISC-IV)
**Case T**<br>$G_2P_2A_0$, Singleton<br>Term<br>Female<br>L Hemiplegic CP<br>GMFCS I	-Family hx of early heart attack	-PV bleeding: 3rd trimester<br>-Gestational diabetes<br>-Medication use: Insulin	-Meconium staining<br>-Shoulder dystocia<br>-APGAR 1min: 1<br>-APGAR 5min: 6	MCA - DM1<br>MCA - DB	38	-Normal intellectual development (based on WPPSI-IV)
**Case U**<br>$G_1P_1A_0$, Singleton<br>Term<br>Female<br>L Hemiplegic CP<br>GMFCS I	-Family hx of early heart attack	-Medication use: type unknown	-Acidosis (pH=7.27)	PVI - V	63	-Normal learning profile (as identified by a developmental pediatrician)
**Case V**<br>$G_5P_4A_1$, Singleton<br>Premature<br>Female<br>L Hemiplegic CP<br>GMFCS I	-Maternal hx of early heart attack	-Toxic exposure: tobacco use	-Seizures at birth	No image	79	-Intellectual disability
**Case W**<br>$G_4P_4A_0$, Singleton<br>Term	-Advanced maternal age (37yrs)<br>-Family hx of early heart attack and clots	-Toxic exposure: tobacco use	-Acidosis (pH=7.3)	PVI - V	66	-Normal intellectual development (based on WISC-IV)

Table S1. Hemi-NET CNV cases with positive clinical risk factors

Female<br>R hemiplegic CP<br>GMFCS I						

%ile: percentile; ABGs: arterial blood gases; abx: antibiotics; ADHD: attention deficit hyperactivity disorder; ASD: autism spectrum disorder; BW: birth weight; CP: cerebral palsy; C-section: cesarean section; decels: decelerations; dx: diagnosis; ETOH: alcohol; FHR: fetal heart rate; FTP: failure to progress; GA: gestational age; GBS: group B streptococcus; $G_nP_nA_n$: gravida/para/abortus; hx: history; IVF: in vitro fertilization; L: left; LGA: large for gestational age; MCA: middle cerebral artery; N/A: not available; NEC: necrotizing enterocolitis; PIH: pregnancy induced hypertension; PROM: prolonged rupture of membranes; PV: per vaginum; R: right; RF: risk factor; SGA: small for gestational age; SVD: spontaneous vaginal delivery; u/s: ultrasoundwks: weeks; yrs: years.

Neuroimaging: **MCA - PM1:** PROXIMAL MCA OR PROXIMAL M1; **MCA – DM1:** DISTAL M1; **MCA – AT:** ANTERIOR TRUNK; **MCA – PT:** POSTERIOR TRUNK; **MCA –MLS**: MEDIAL LENTICULOSTRIATE; **MCA – LLS:** LATERAL LENTICULOSTRIATE; **MCA – DB:** DISTAL MCA BRANCH; **PVI – V:** PERIVENTRICULAR VENOUS INFARCTION; **PV – GM:** PERIVENTRICULAR GERMINAL MATRIX INJURY.
