# Supplementary material for: De novo and rare inherited copy-number variations in the hemiplegic form of cerebral palsy
Source: Genet Med. 2017 Aug 3;20(2):172–80. doi: 10.1038/gim.2017.83 (PMC5846809; doi:10.1038/gim.2017.83)
Supplement: Supplementary Information [file gim201783x5.pdf]

Supplementary information for

De novo and rare inherited copy number variations in the hemiplegic form of cerebral palsy

Mehdi Zarrei, PhD[1], Darcy L. Fehlings, MD[2], Karizma Mawjee, MA[2], Lauren Switzer, MSc[2], Bhooma Thiruvahindrapuram, MSc[1], Susan Walker, PhD[1], Daniele Merico, PhD[1,3], Guillermo Casallo, BSc[1], Mohammed Uddin, PhD[1], Jeffrey R. MacDonald, BSc[1], Matthew J. Gazzellone, MSc[1], Edward J. Higginbotham, BSc[1], Craig Campbell, MD[4], Gabrielle deVeber, MD[5], Pam Frid, MD[6], Jan Willem Gorter, MD[7], Carolyn Hunt, MD[8], Anne Kawamura, MD[2], Marie Kim, MD[9], Anna McCormick, MD[10], Ronit Mesterman, MD[7], Dawa Samdup, MD[11], Christian R. Marshall, PhD[1], Dimitri J. Stavropoulos, PhD[12], Richard F. Wintle, PhD[1], Stephen W. Scherer, PhD[1,13]

The first two authors contributed equally to this work.

[1]The Centre for Applied Genomics and Program in Genetics and Genome Biology, The Hospital for Sick Children, Toronto, Ontario, Canada; [2]Holland Bloorview Kids Rehabilitation Hospital, Department of Paediatrics, University of Toronto, Toronto, Ontario, Canada; [3]Deep Genomics Inc., Toronto, Ontario, Canada; [4]Department of Pediatrics, Schulich School of Medicine, Western University, London, Ontario, Canada; [5]Division of Neurology, Department of Pediatrics, Hospital for Sick Children, University of Toronto, Toronto, Ontario, Canada; [6]Thames Valley Children's Centre, London, Ontario, Canada; [7]McMaster University, Hamilton, Ontario, Canada; [8]Grandview Children's Centre, Oshawa, Ontario, Canada; [9]ErinoakKids Centre for Treatment and Development, Mississauga, Ontario, Canada; [10]Ottawa Children's Treatment Centre, Ottawa, Ontario, Canada; [11]Hotel Dieu Hospital, Kingston, Ontario, Canada; [12]Department of Pediatric Laboratory Medicine, Genome Diagnostics, The Hospital for Sick Children, Toronto, Ontario, Canada; [13]Department of Molecular Genetics and McLaughlin Centre, University of Toronto, Toronto, Ontario, Canada. Correspondence: Stephen W. Scherer (stephen.scherer@sickkids.ca)

December 23, 2016; revised June 06, 2017

Participants

We submitted DNA extracted from saliva from 103 trio-based families to The Centre for Applied Genomics (Toronto, Canada). Of these, 101 families including 292 individuals passed the quality control steps. Of these, 91 families were trios; six families failed quality control. In our final analysis, for probands for whom we had CNVs for only one parent, if a CNV was present in only the patient, we tested the inheritance in the missing parent's DNA using complementary methods (see below). We, therefore, analyzed 97 probands for the current study. However, we presented rare CNV calls for all subjects that passed the initial quality control.

Table S1 A summary of risk factors for CP in selected cases (CNV positive group) with a risk factor gene [see online Table S1.docx]

Variant calling and rare variant detection

We called CNVs as previously described.[1] Briefly, four different CNV calling algorithms were used to generate high-confidence CNVs. These included the Affymetrix Chromosome Analysis Suite (ChAS), iPattern, BioDiscovery Nexus, and Partek Genomics Suite. "Stringent" CNVs were defined for further analyses. This set included CNVs detected by one or both of ChAS or iPattern, and if detected by only one of these, then also by one of Nexus or Partek. For stringent calls on the X chromosome, we required calling by both ChAS and iPattern. We inspected Y chromosome CNVs, but found nothing potentially relevant to CP, and did not report them in our downstream analysis.

Rare variants were defined as those present in < 0.1% of our population controls, using a 50% reciprocal overlap threshold, considering deletion and duplication matched variants.[2,3] The rare variants were required to be called by a minimum of 10 successive probes, have a size of greater than 10 kb, and have less than 70% of their length overlapped by segmental duplications (Figure S1). We used an additional filtering step to eliminate potential artifacts, which removed any variant that was in a region less than 75% copy number stable, according the stringent CNV map of the human genome.[2] We used eight population control cohorts with a total of 10,851 unrelated European and Non-European subjects in the current study. PoPGen (Population-Based Recruitment for Genetics Research; n=1,107)[4] and OHI (Ottawa Heart Institute controls; n=1,224)[5] samples were genotyped using the Affymetrix Genome-Wide Human SNP Array 6.0 platform. KORA (Cooperative Health Research in the Region of Augsburg; n=1,775)[6] and COGEND (Collaborative Genetic Study of Nicotine Dependence; n=1,109)[7] samples were genotyped using the OMNI 2.5M quad array platform. SAGE consortium (Study of Addiction: Genetics and Environment; n=1,764),[8] ONC (Ontario Familial Colorectal Cancer Registry; n=433),[9] and HABC (Health, Aging, and Body Composition study; n=2,566)[10] were genotyped using the Illumina Human1M microarray. A total of 873 samples from the Ontario Population Genomics Platform (OPGP) population control database[1] genotyped using the Affymetrix CytoScan HD array were also used.

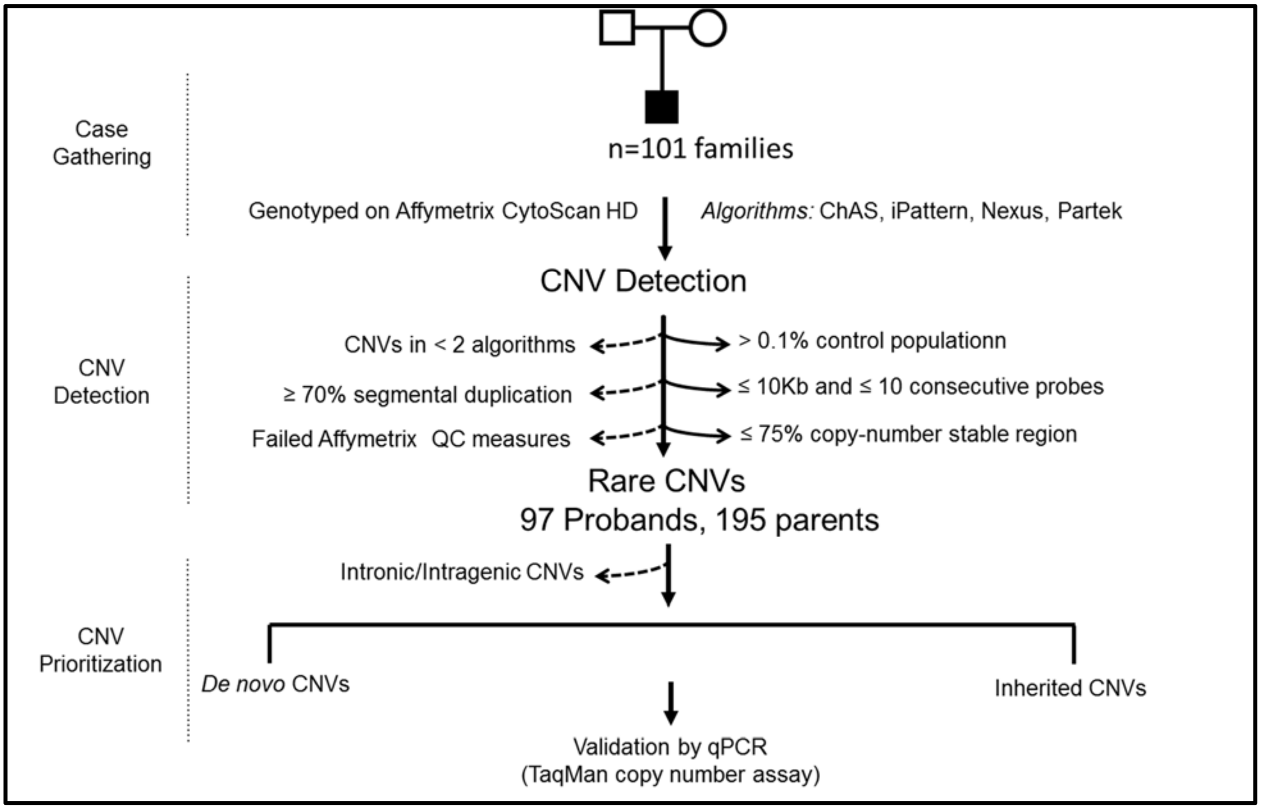

Figure S1 Pipeline used for CNV analysis

Table S2: Rare variant calls in samples used for the current analysis, with sex, ancestry and family status [see online Table S2.xlsx]

Validation of CNV findings

The potentially clinically relevant CNVs were validated in the proband and parental DNA samples using a SYBR Green-based real-time quantitative PCR assay, TaqMan Copy number assays, or droplet digital PCR. Control primers were designed to amplify region of the *FOXP2* gene. NA10851 and NA15510 were used as our male and female controls, respectively. Primer sequences used to amplify candidate regions and TaqMan probes' IDs are presented in Table S3**.**

Table S3 SYBR and TaqMan probes were used for secondary confirmation for the CNVs in the current study [see online Table S3.xlsx]

Critical exons

To compute the burden of rare missense and loss-of-function mutations for each exon, we used whole-genome sequencing data from the 1,000 Genomes Project;[11] exon level expression data from RNA sequencing were obtained for 524 brain tissues (prenatal and postnatal postmortem donors) from the BrainSpan project.[12] To classify critical exons, we computed the 75th percentile of brain expression and mutational burden for each exon as described.[13,14] For the current analysis, we used all genes whose coding sequences were impacted by rare CNVs (size < 2 Mb). We used two sets of controls: (i) a platform matched collection of 873 samples genotyped on the Affymetrix CytoScan HD platform, and (ii) all 10,851 control samples mentioned above. After removing the duplicated genes, we used 198 vs. 927 genes, in cases and controls, respectively, for the first set, and 81 vs. 6,644 genes in cases and controls for the second set. For each set of genes, we first computed the fraction of critical exons for each brain tissue (16 regions; see Figure 1 legend). Next, we subdivided the brain tissue regions according to developmental stage: prenatal (12-37 weeks of gestation), early postnatal and adolescence (4 months-15 years) and adult (> 18 years). The fraction of critical exons for each stage was then compared between case and control genes impacted by CNVs. We conducted a T-test on the difference of the critical exon fraction between the two groups (case-control) for each developmental stage and brain region.

Cases with notable CNV findings

We grouped potentially clinically-relevant CNVs into two main categories: i) *de novo* and ii) inherited. Both groups included variants known to be associated with curated DECIPHER syndromes. We did a thorough literature search for each CNV and provided for each a description of why it may be relevant to CP, accompanied by a map of genes impacted and the CNV map of the human genome.[2] The descriptions of the CNVs are provided in the main text and in this document.

A: *De novo* CNVS, CNVs known to be associated with curated DECIPHER syndromes, or those inherited from parents and referred to in the main text

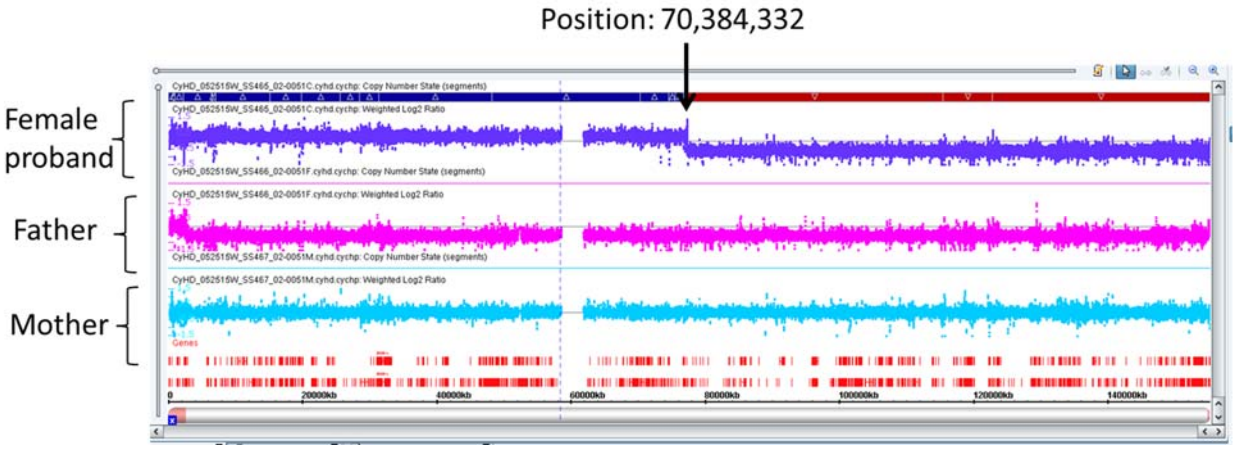

Figure S2 A *de novo* deletion and duplication in case B.

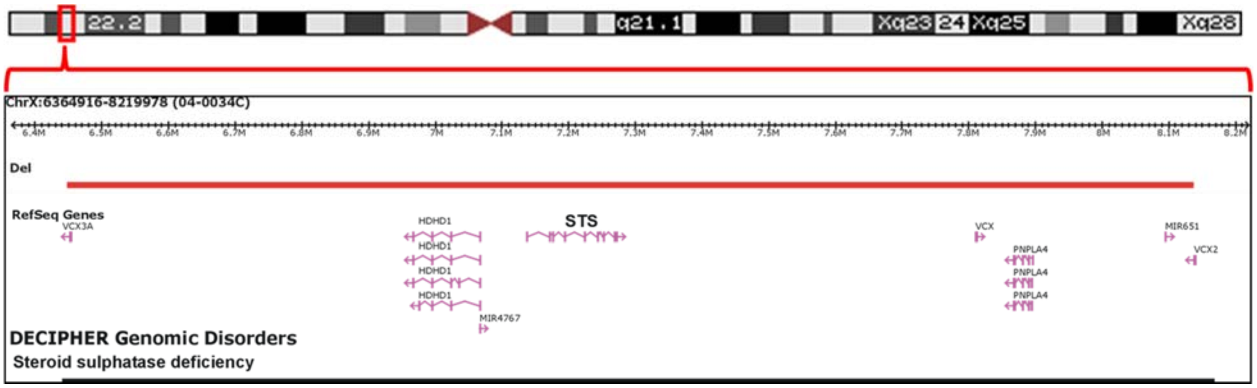

Figure S3 A 1.68 Mb *de novo* deletion congruent with steroid sulphatase deficiency syndrome in a female patient (case C).

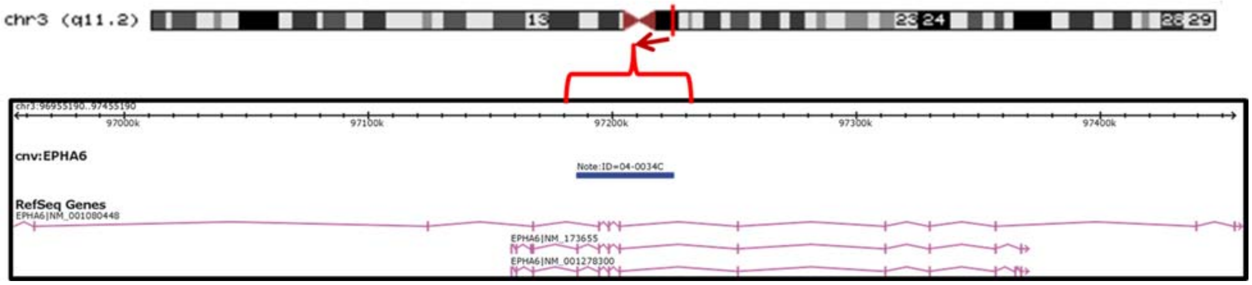

Figure S4 A 39.6 Kb maternally inherited duplication impacting the *EPHA6* gene in case C.

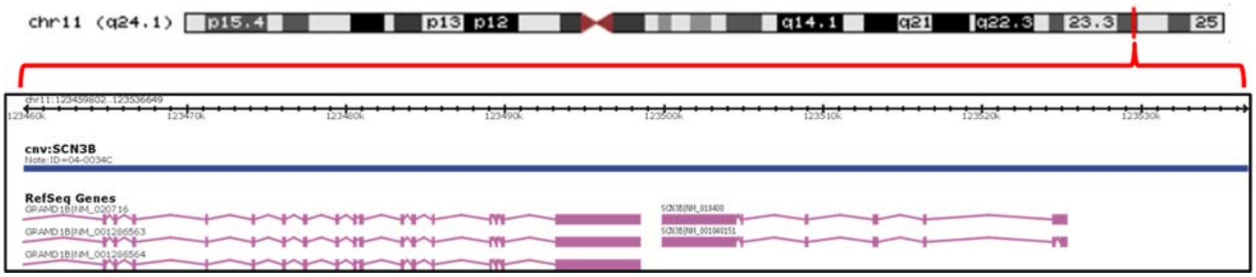

Figure S5 A 76.8 Kb maternally inherited duplication impacting the *SCN3B* and *GRAMD1B* genes in case C.

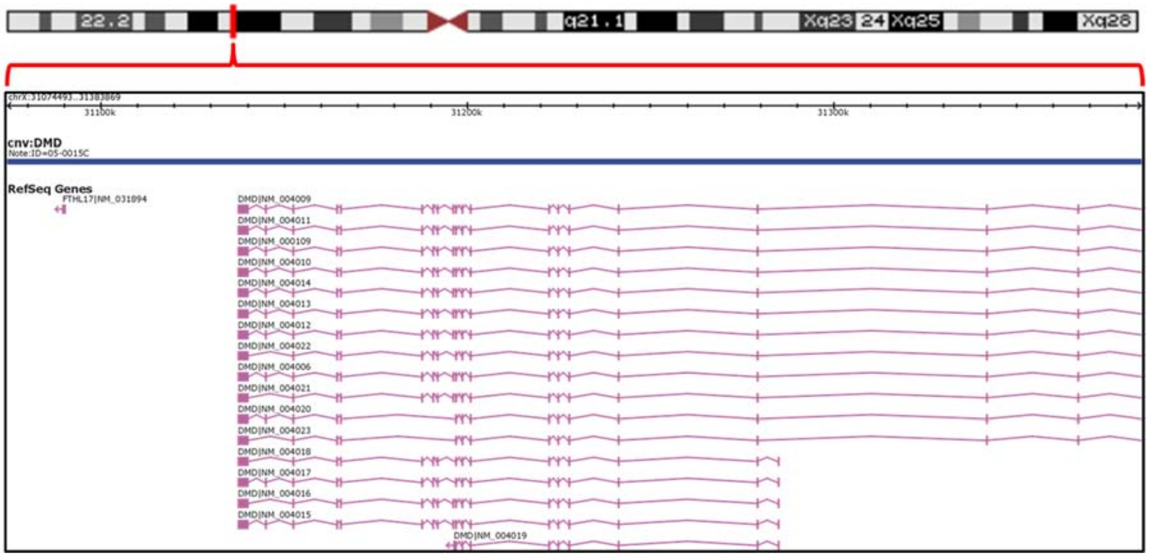

Figure S6 A 309.4 Kb *de novo* duplication impacting the *DMD* and *FTHL17* genes in case D.

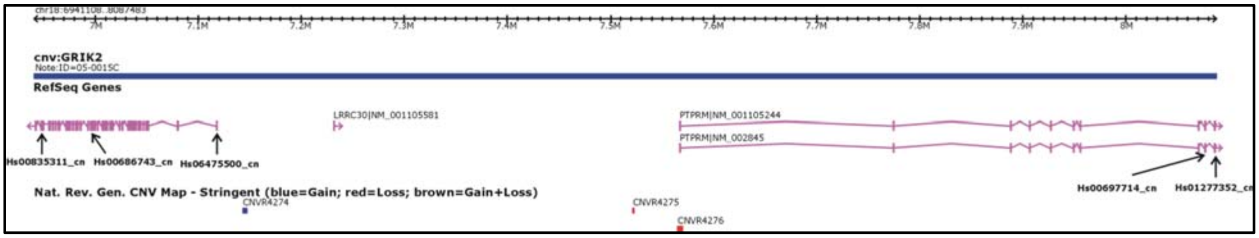

Figure S7 A 1.15 Mb *de novo* duplication impacting the *LAMA1*, *PTPRM*, and *LRRC30* genes in case D. TaqMan probes used for confirmation of this CNV are shown. The *LAMA1* gene is not duplicated entirely. The last exon was not affected, confirmed by TaqMan probes.

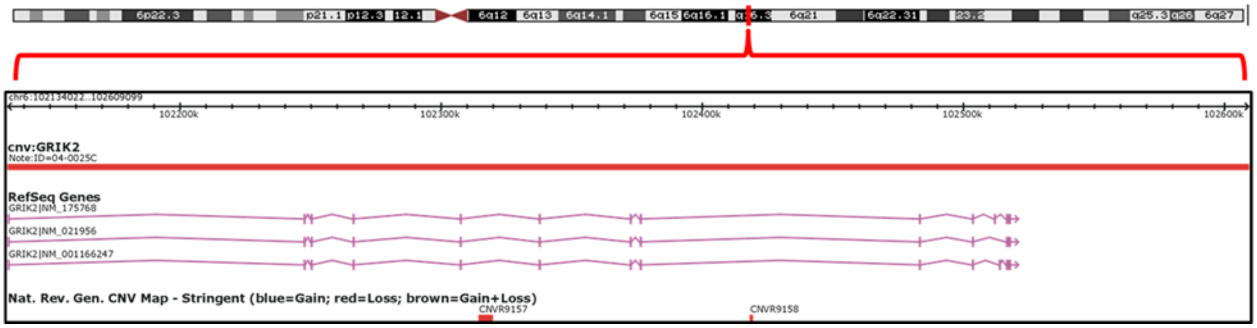

Figure S8 A 475.1 Kb *de novo* deletion impacting the *GRIK2* gene in case E.

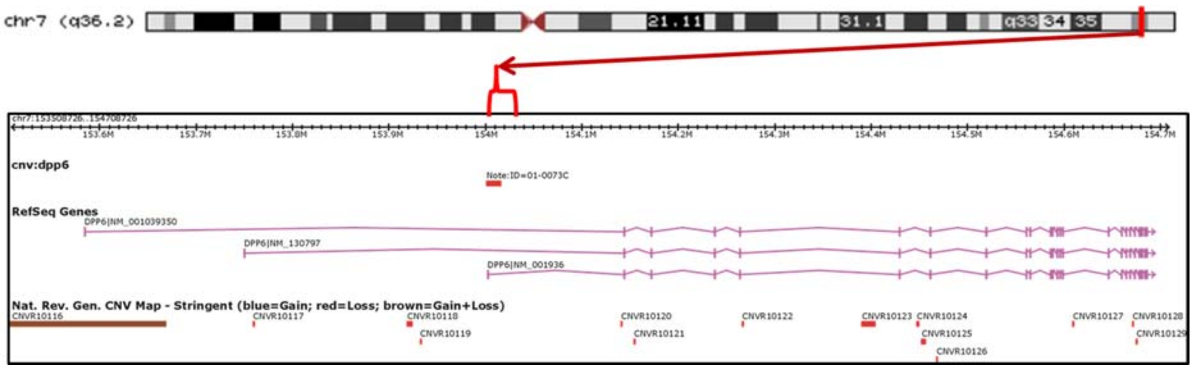

Figure S9 A 14.6 Kb paternally inherited deletion impacting the *DPP6* gene in case G.

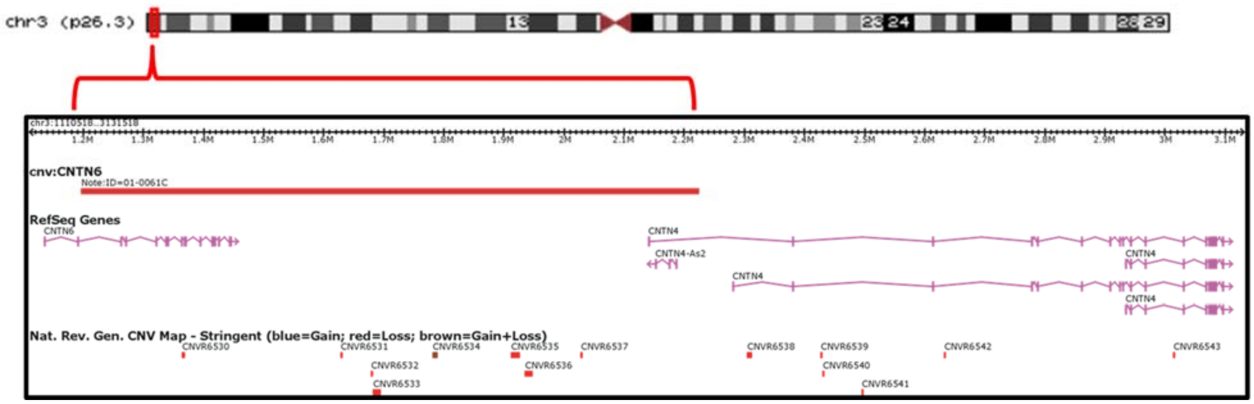

Figure S10 A 1.03 Mb paternally inherited deletion impacting *CNTN6* and *CNTN4* in case I.

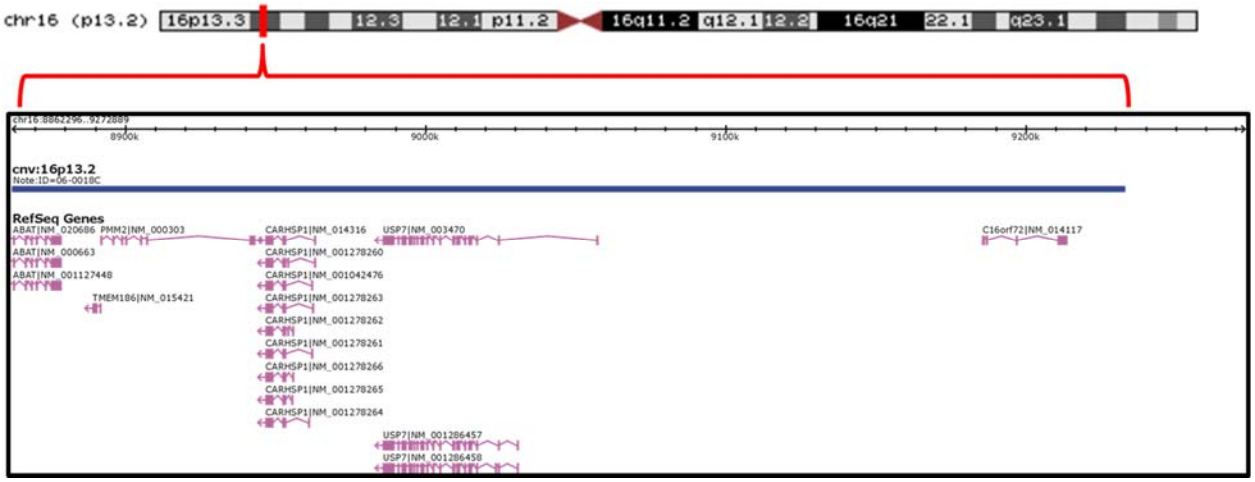

Figure S11 A 370.6 Kb paternally inherited duplication in case L.

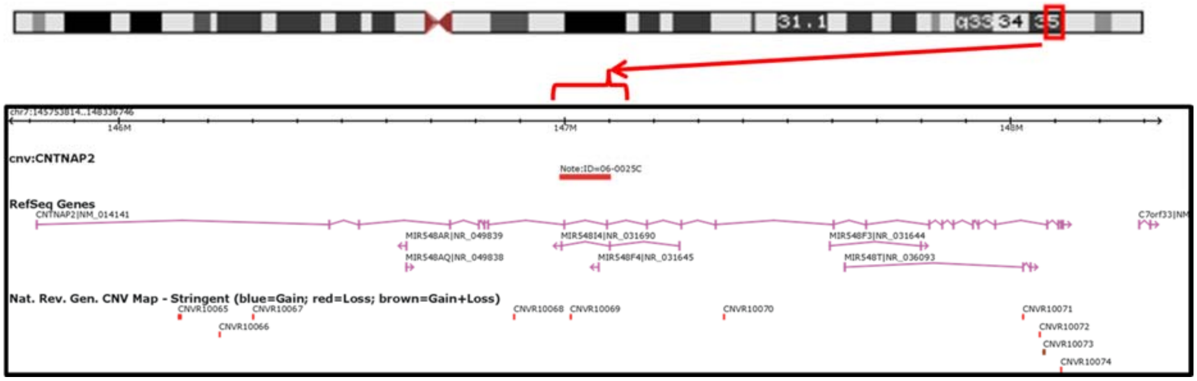

Figure S12 A 114.4 Kb maternally inherited deletion impacting the *CNTNAP2* gene in case Q.

Expanding genes impacted by CNVs using GeneMANIA

The list of genes impacted by the rare CNVs presented in Table 1, limited to CNVs of smaller than 2 Mb, was extended with functionally similar genes (100 genes) using GeneMANIA[15] (Figure S13).

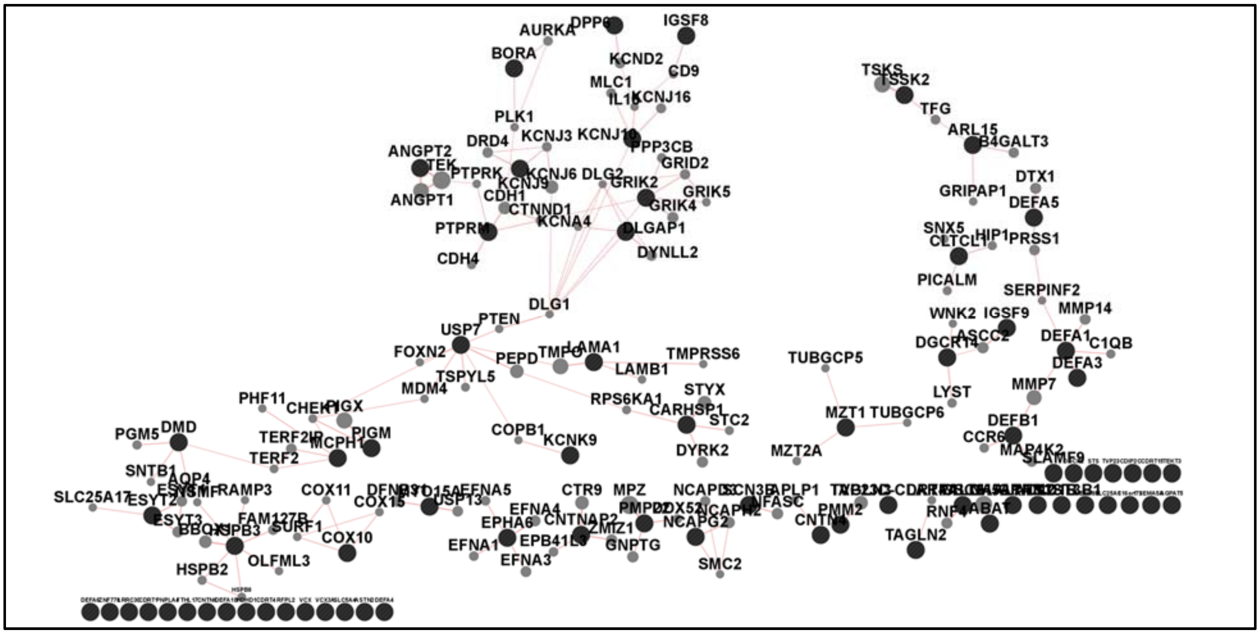

Figure S13 Genes expanded with functionally similar genes using GeneMANIA.

B: Inherited CNVS impacting neurodevelopmental or muscular function genes that are not referred to in the main text

Case J is a male harboring a 973 kb duplication which impacts the *MCPH1*, microcephalin 1 gene (Figure S14). Abnormal function of this gene, due to partial deletion of exons or single nucleotide mutations leading to loss-of-function or truncation of its protein products, is linked to the autosomal recessive primary microcephaly and intellectual disability.[16,17] Moreover, both deletion and duplication of this gene have been previously linked to ASD in patients with fine and gross motor skills which were normal or in severe mental retardation.[18,19] This CNV duplicates the entire gene, which is expressed in the developing cerebral cortex of the fetal brain, is involved in the development of the cerebral cortex and regulation of cerebral cortex size, and may act by increasing the dosage of the gene products. No duplication of this gene has been previously linked to microcephaly. A duplication involving the first exon of this gene is reported in a patient with cerebral palsy.[20]

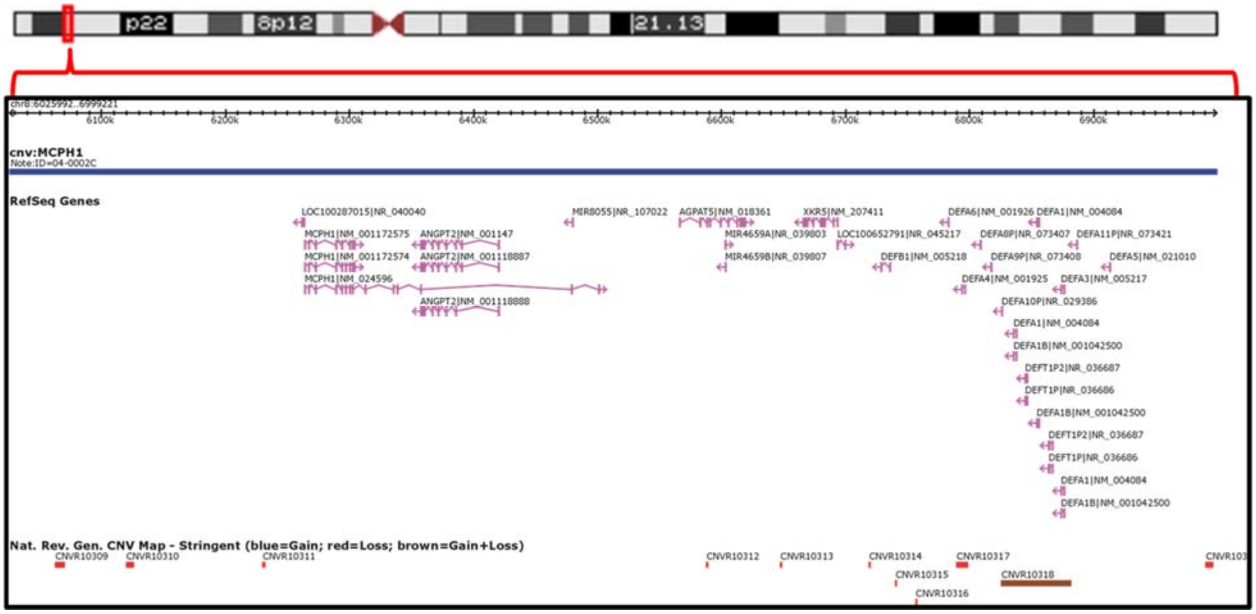

Figure S14 A 973.2 kb maternally inherited duplication case J.

Case K is a female with a maternally inherited 504.6 kb deletion impacting the *HSPB3* gene (Figure S15). Missense mutations in this gene, which encode a muscle specific small heat-shock protein with a function in muscle maintenance, are associated with motor neuropathy.[21,22]

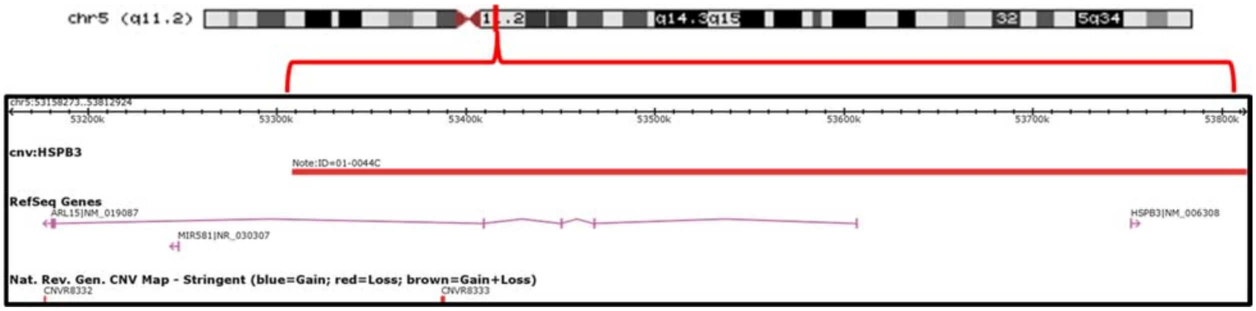

Figure S15 A 504.6 kb maternally inherited duplication impacting the *HSPB3* and *ARL15* genes in case K.

Case M is a male with two maternally inherited duplications. The first one is a 255.8Kb CNV impacting the *KCNK9* gene (Figure S16). Proper K^+ channels, encoded by *KCNK9*, might have a neuroprotective role in ischemic stroke.[23-25] Disrupted K^+ channels might, therefore, increase the brain damage in patients who encountered hypoxia during delivery, resulting in cerebral palsy. A possible link between the late onset of seizures and mutations in *KCNK9* is established.[26] Mutations that alter the function of the expressed maternal copy have been identified as the cause of Birk-Barel Mental Retardation Dysmorphism Syndrome.[27] Affected patients present with severe mental retardation, hyperactivity, hypotonia and weakness of the proximal muscles.[27] A mutation in another potassium ion channel, *KCNC3*, has been identified in a patient with ataxic cerebral palsy.[28] The second duplication is a 251 kb duplication, impacting all four isoforms of the *NCAPG2* gene and the *ESYT2* gene (Figure S17). Deletions of both genes have been proposed to contribute to mental deficiency and severe microcephaly phenotypes.[19] However, no duplication has been reportedly linked to any disease for those genes. This patient has language-based learning disability.

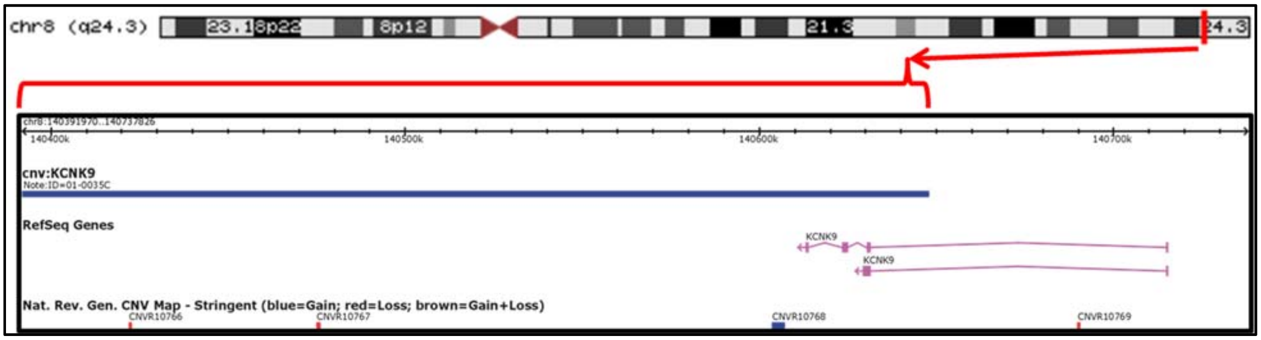

Figure S16 A 255.8 Kb maternally inherited duplication impacting the *KCNK9* gene in case M.

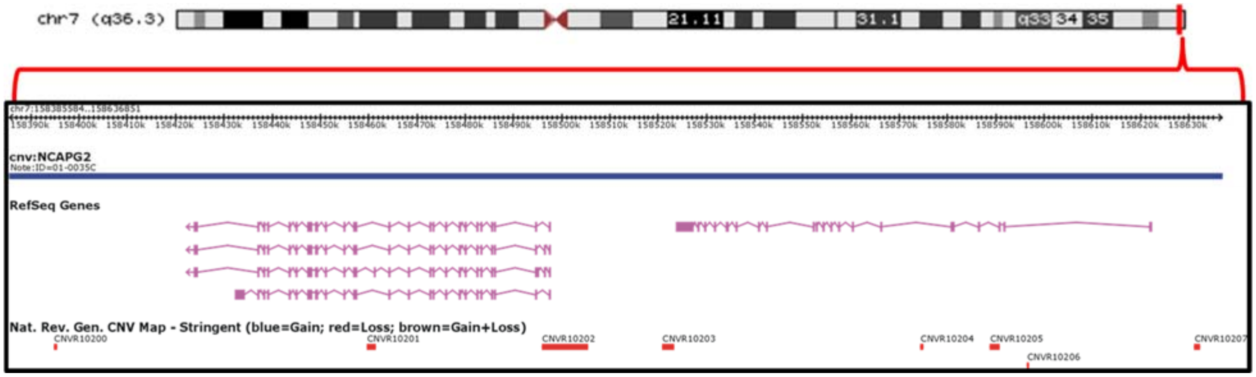

Figure S17 A 255 kb maternally inherited duplication in case M.

Case N is a female carrying a 196.3 kb duplication impacting seven genes including the *KCNJ10, KCNJ9* and *IGSF9* genes (Figure S18). *KCNJ10, KCNJ9* encode a member of the potassium channel family and may have a function in the potassium buffering actions of glial cells in the brain. It has a critical role in the functioning of the central nervous system.[29] SNP variations in this gene have been linked to congenital hearing loss.[30,31] *KCNJ10* gene mutations also cause EAST syndrome, i.e. epilepsy, ataxia, sensorineural deafness, and tubulopathy.[32,33] *KCNJ9* (*GIRK3*) is another member of the potassium channel family, and a function in the mesolimbic dopaminergic pathway and γ-aminobutyric acid type B (GABAB) receptors.[34,35] Mouse model studies on another gene impacted by this CNV, *IGSF9* which is an immunoglobulin superfamily gene member, has been implicated in the outgrowth and branching of neurites, exon guidance, and synapse maturation.[36] This gene has a role in the inhibitory synapse development and maintenance.[37]

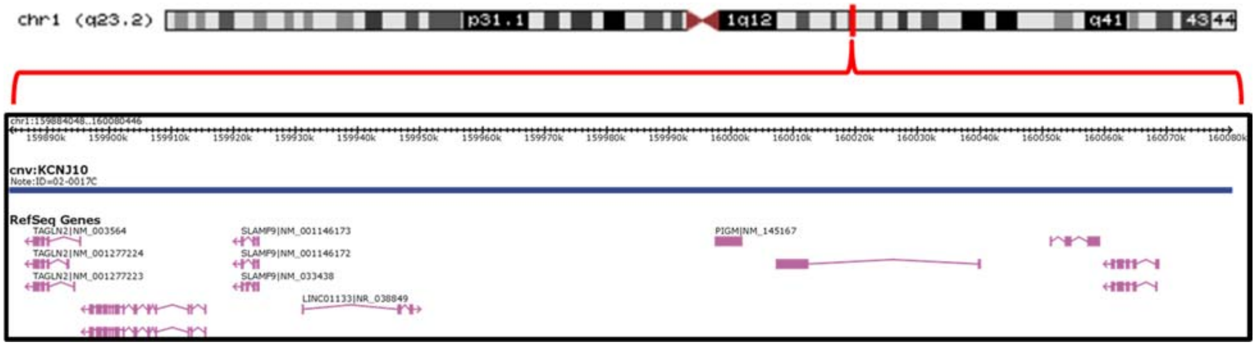

Figure S18 A 196.3 kb maternally inherited duplication in case N.

Case O carries a maternally inherited 176.4 kb deletion impacting two exons of the *NAALADL2* gene (Figure S19). Deletion of the entire gene, inherited from the healthy mother, is reported in a case with microcephaly, seizure, and severe intellectual disability.[38] It has been implicated in developmental malformations.[38] A sibling of this patient has a maternally inherited CNV impacting *NAALADL2* similar to Case O.

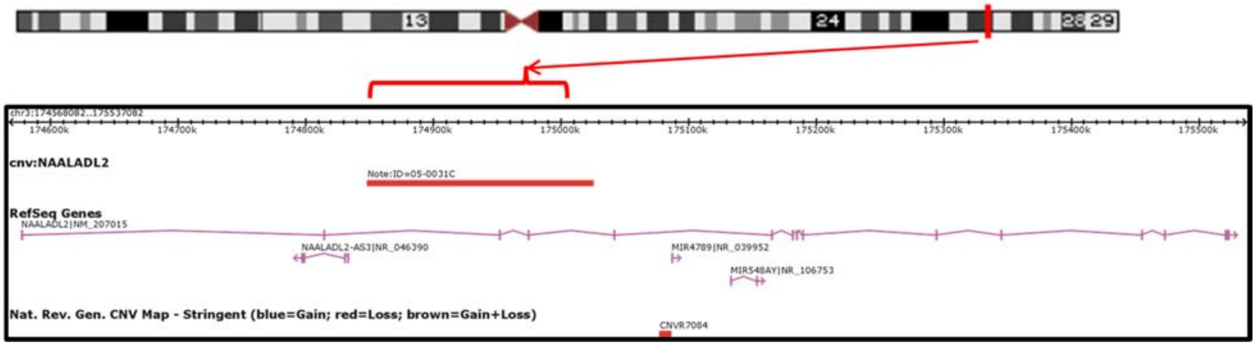

Figure S19 A 176.4 kb maternally inherited deletion impacting the *NAALADL2* gene in case O.

Case P is a female carrying a 162.4 kb duplication nested in the 22q11.2 deletion locus (Figure S20). Previous studies reported an association between variations in *DGCR2* and *DGCR14* genes, two of the genes duplicated by this CNV, and schizophrenia.[39,40] Our patient has ADHD, ASD, and had a seizure on day four of her life (Table S1).

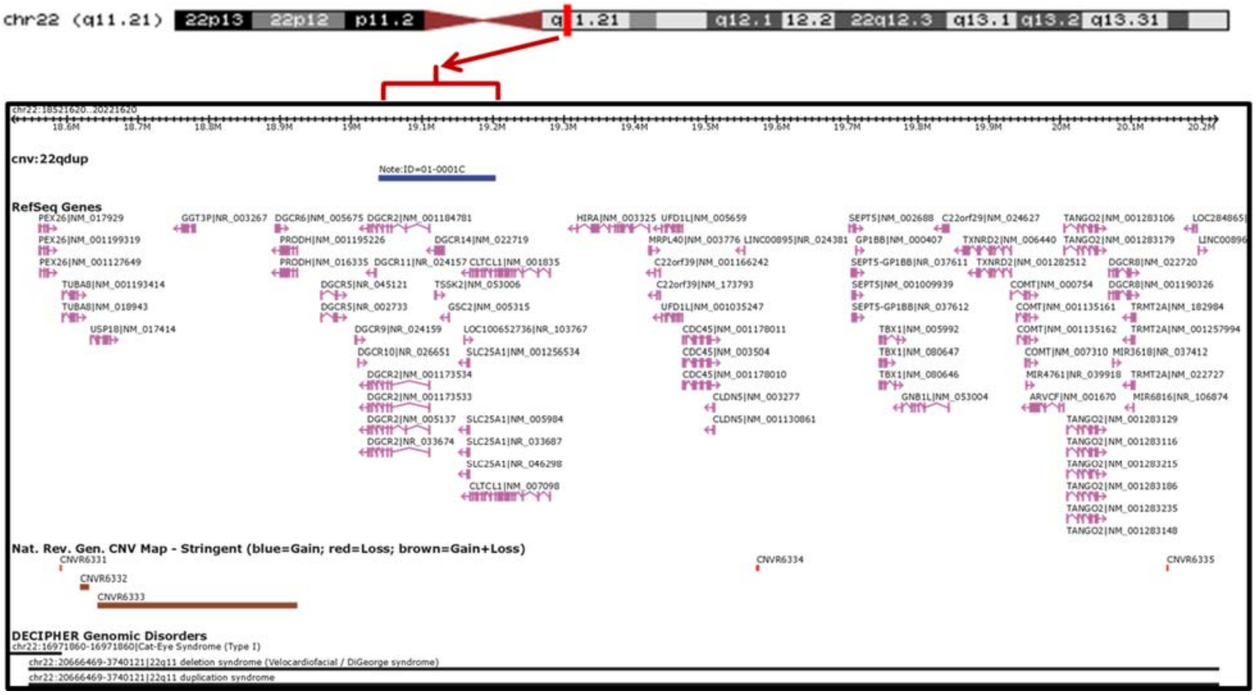

Figure S20 A 162.4 kb maternally inherited duplication case P.

Case R is a female carrying a maternally inherited 77.5 kb deletion impacting seven exons of the *SEMA5A* gene (Figure S21). *SEMA5A* acts as a bi-functional guidance cue, having both attractive and inhibitory effects on developing axons.[41] A deletion encompassing the end of this gene has been reported in a boy with ASD and intellectual disability.[41] This gene is also associated with hippocampal volume and Parkinson's disease.[42,43] This patient has a language-based learning disability.

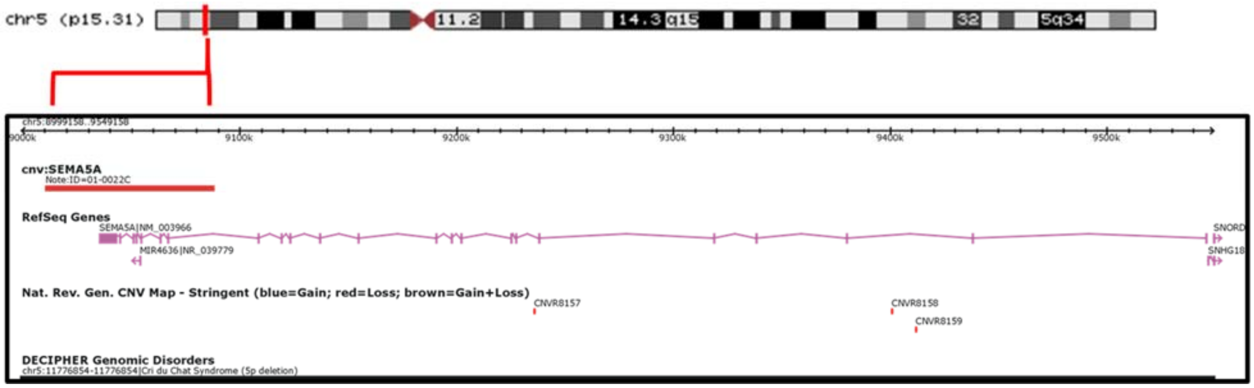

Figure S21 A 77.5 kb maternally inherited deletion impacting the *SEMA5A* gene in case R.

Case S is a female carrying a 40.8 kb paternally inherited duplication impacting the forth exon of the *ASTN2* gene (Figure S22). The astrotactin 2 gene is expressed in the brain and may function in neuronal migration. Deletion of this gene has been linked to ASD and ADHD.[44,45] There is a single case with a deletion in the fourth exon of *ASTN2* diagnosed with ASD in our previous publication.[44]

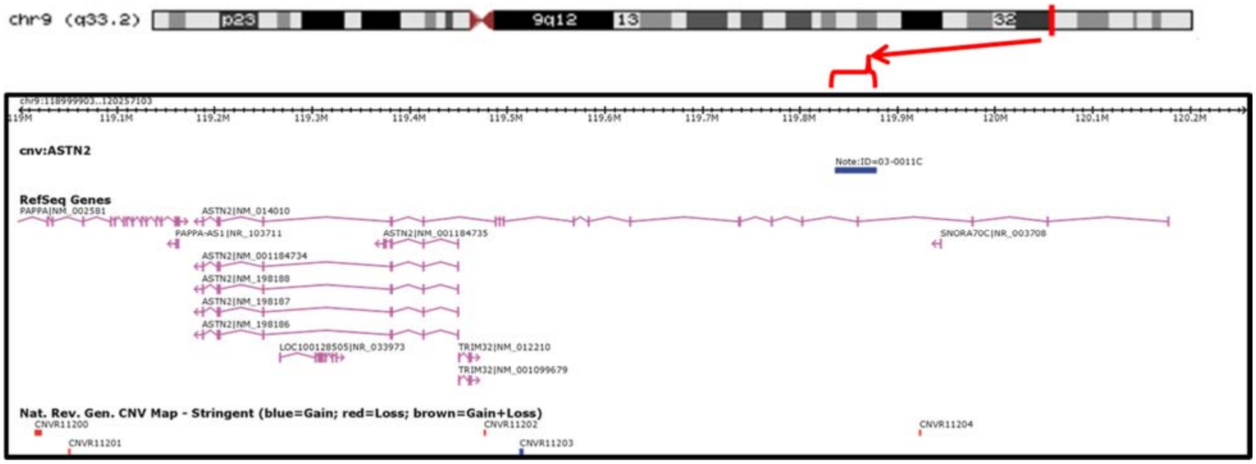

Figure S22 A 40.8 kb paternally inherited duplication in case S.

Case T carries a paternally inherited 34 kb deletion impacting the *RFPL2* and *SLC5A4* genes (Figure S23). *RFPL2* functions in neocortex development.[46]

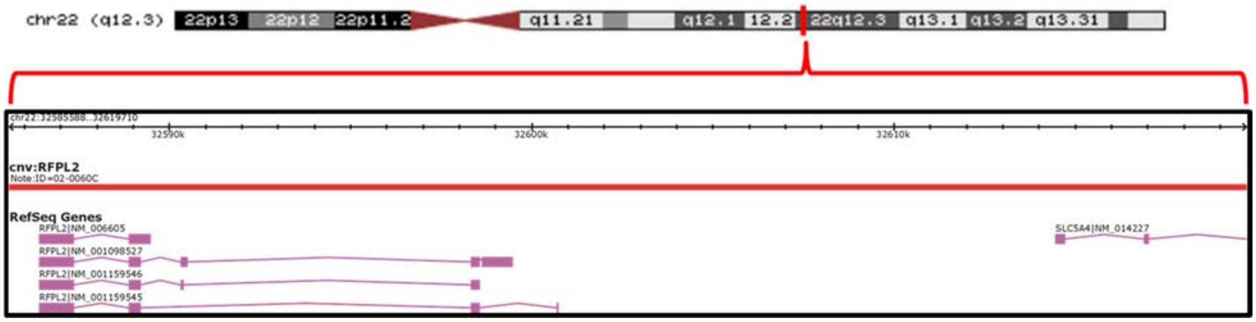

Figure S23 A 34.1 kb paternally inherited deletion impacting the *RFPL2* and *SLC5A4* genes in case T.

Case U is a female carrying a maternally inherited 25.6 kb deletion impacting one exon in the *DLGAP1* gene (Figure S24). Deletions in this gene have been seen in patients diagnosed with Obsessive Compulsive Disorder.[47]

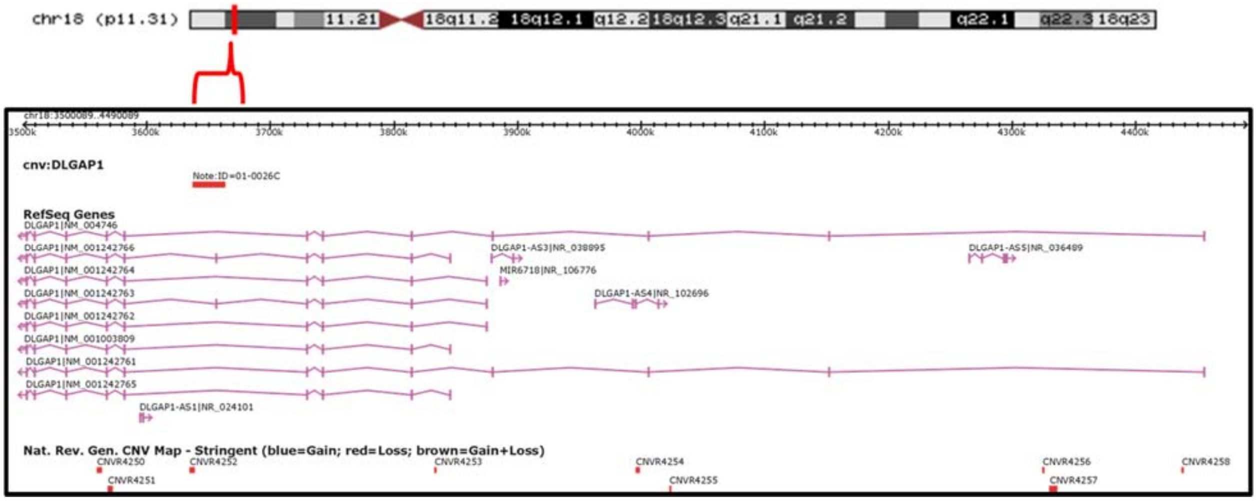

Figure S24 A 25.6 kb maternally inherited deletion impacting the *DLGAP1* gene in case U.

Case V is a female with a maternally inherited 24.1 kb duplication overlapping all exons of the *ZNF778* gene (Figure S25). Haploinsufficiency of this gene has been linked to autism, developmental delay, and variable cognitive impairments of 16q24.3 microdeletion syndrome.[48,49] However, case V harbors a duplication.

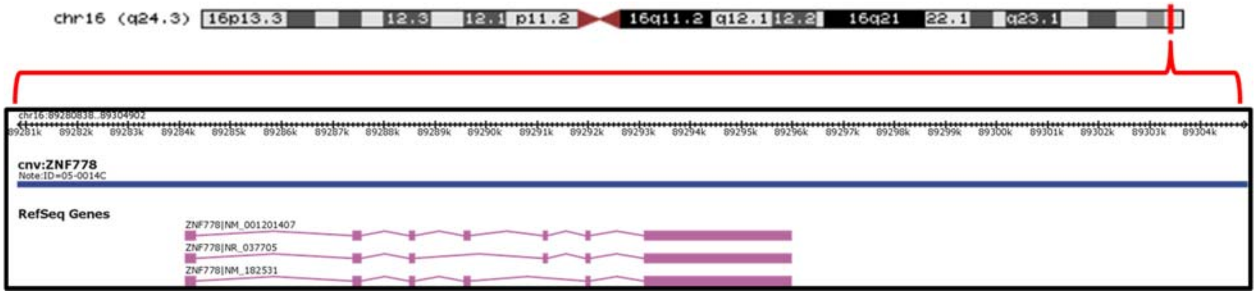

Figure S25 A 24.1 kb maternally inherited duplication impacting the *ZNF778* gene in case V.

Case W is a female carrying a paternally inherited 16.6 kb duplication impacting 15 of the 67 exons of the *MYO15A* gene (Figure S26). Point mutations in this gene have been shown to cause hearing loss in both human and mouse.[50]

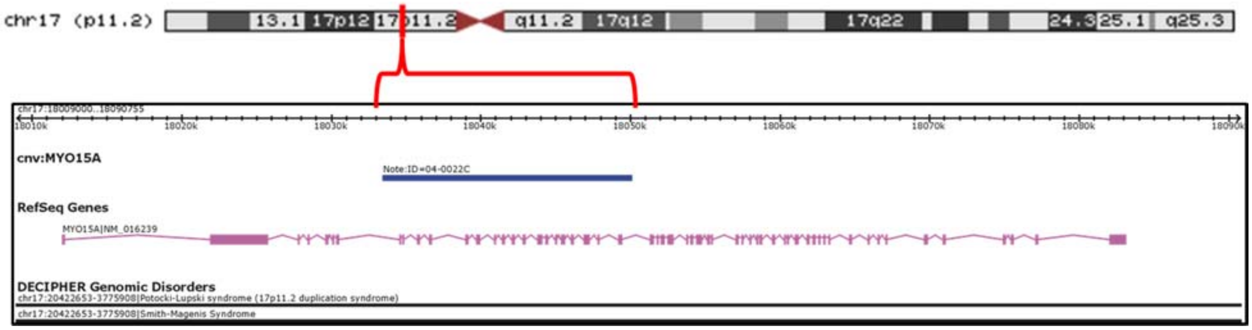

Figure S26 A 16.6 kb paternally inherited duplication impacting the *MYO15A* gene in case W.

Deletions in *DIP2C*:

We found three cases, cases F and G in the current study and 13-019 from our previous publication[51], carrying *de novo* deletions impacting two exons in *DIP2C*. We first performed ddPCR on the probands and their biological parents using eleven different TaqMan assays (Table S3). The results are presented in Figures S27-S29.

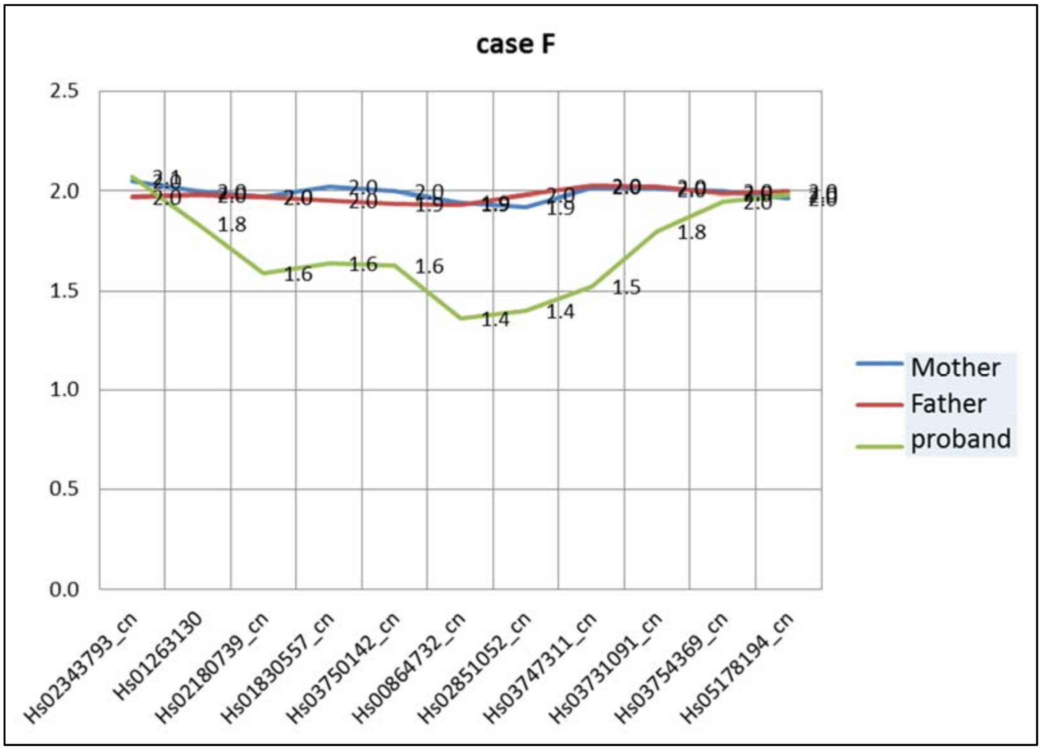

Figure S27 ddPCR results for the deletion impacting *DIP2C* in case F.

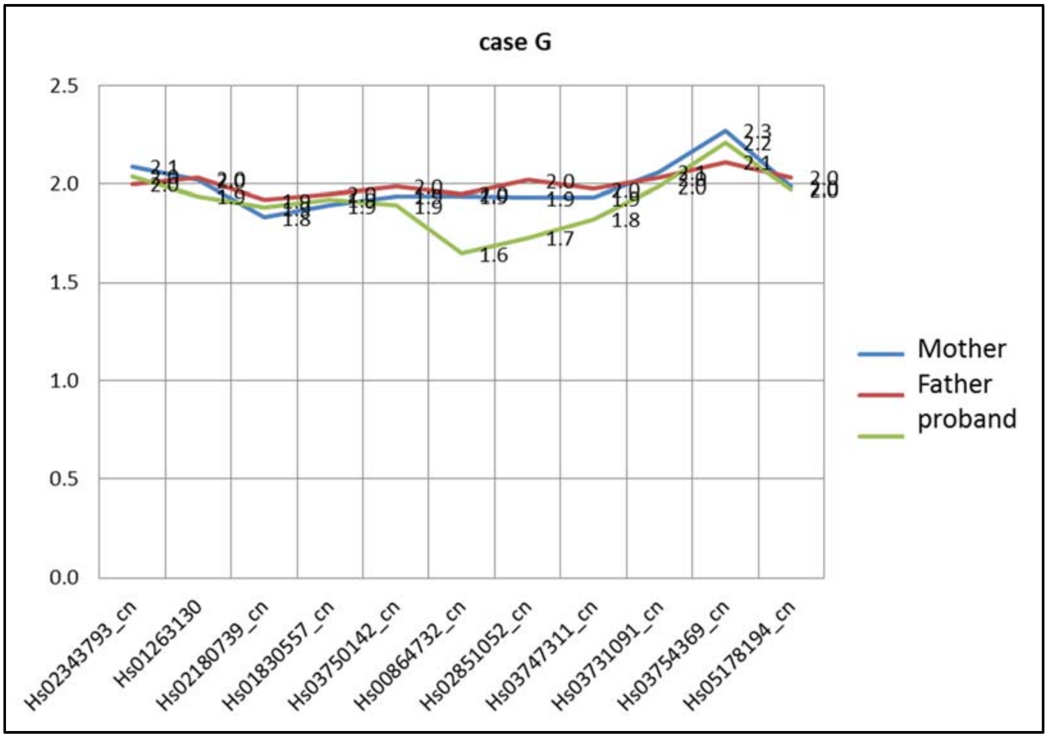

Figure S28 ddPCR results for the deletion impacting *DIP2C* in case G.

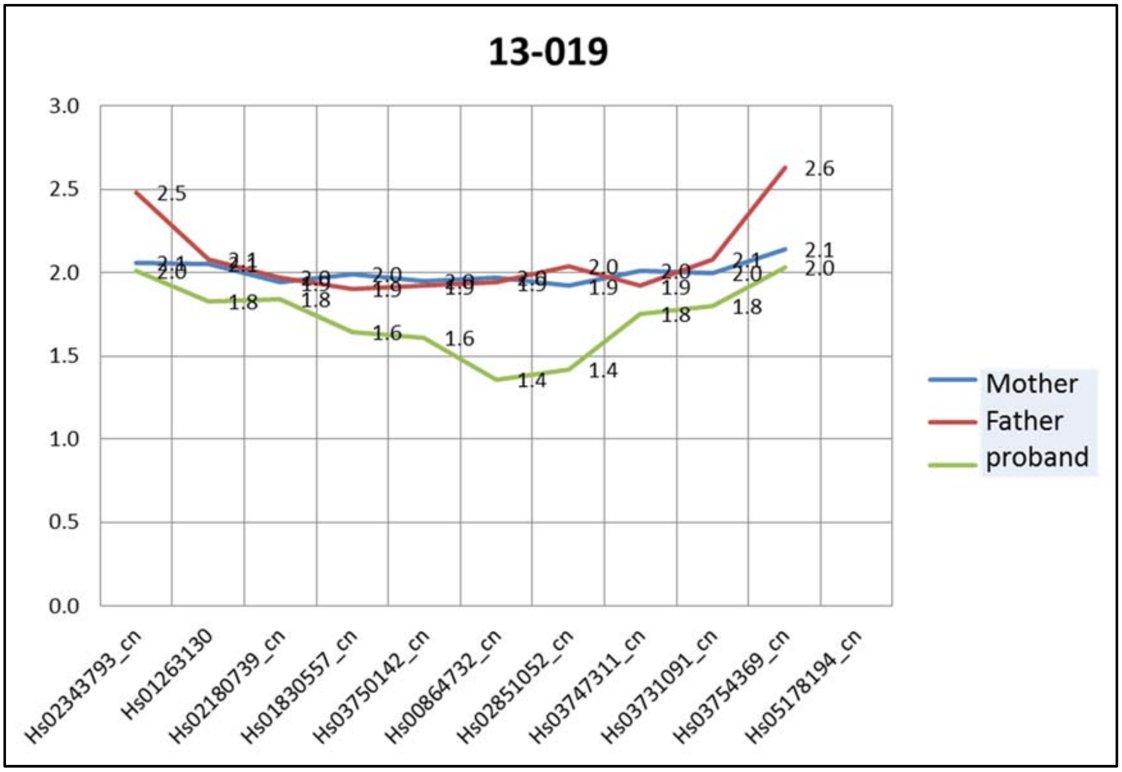

Figure S29 ddPCR results for the deletion impacting *DIP2C* in 13-019.

We also performed a ddPCR using Hs03750142 and Hs02851052 with different color tag. The ratio of the copy numbers between them were plotted in Figure S30.

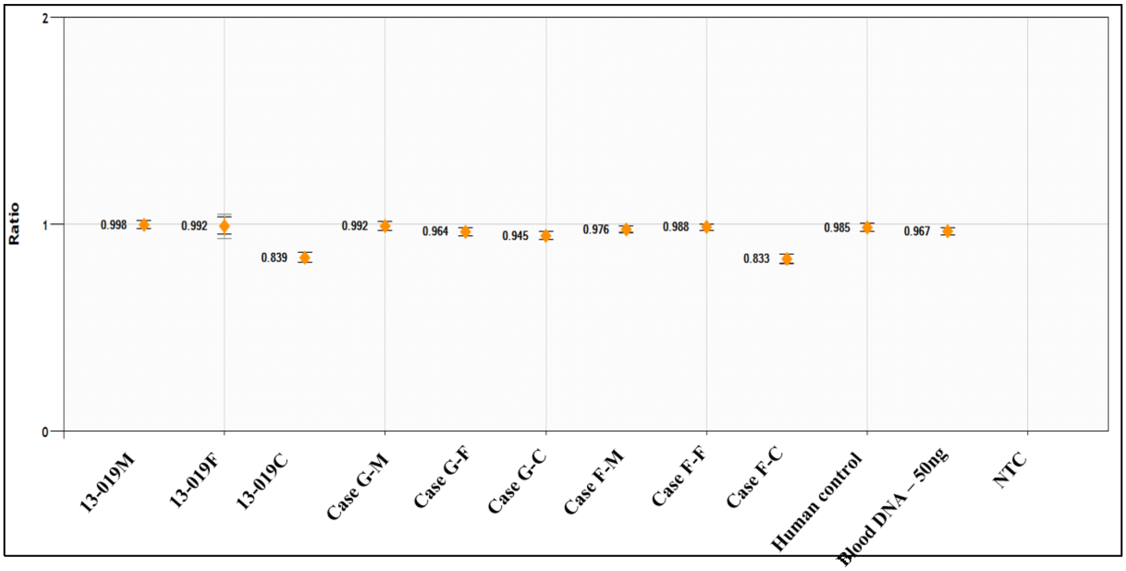

Figure S30 ddPCR results using probes with two different colors.

Critical exons

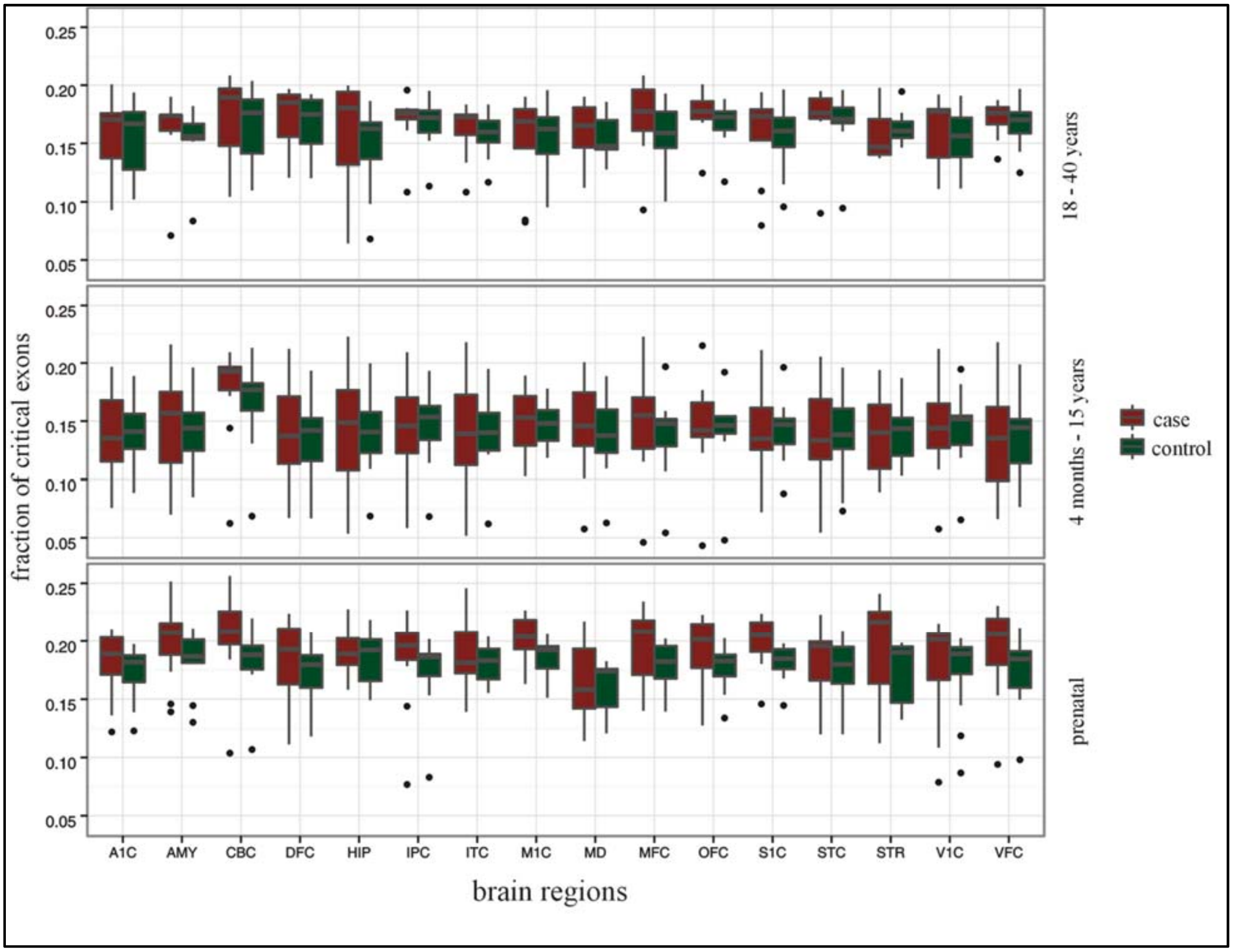

Figure S31 The proportion of critical exons impacted by CNVs in the cases and the 9,611 population controls in 16 different brain regions. The differences were not statistically significant after FDR corrections using Benjamini Hochberg method.

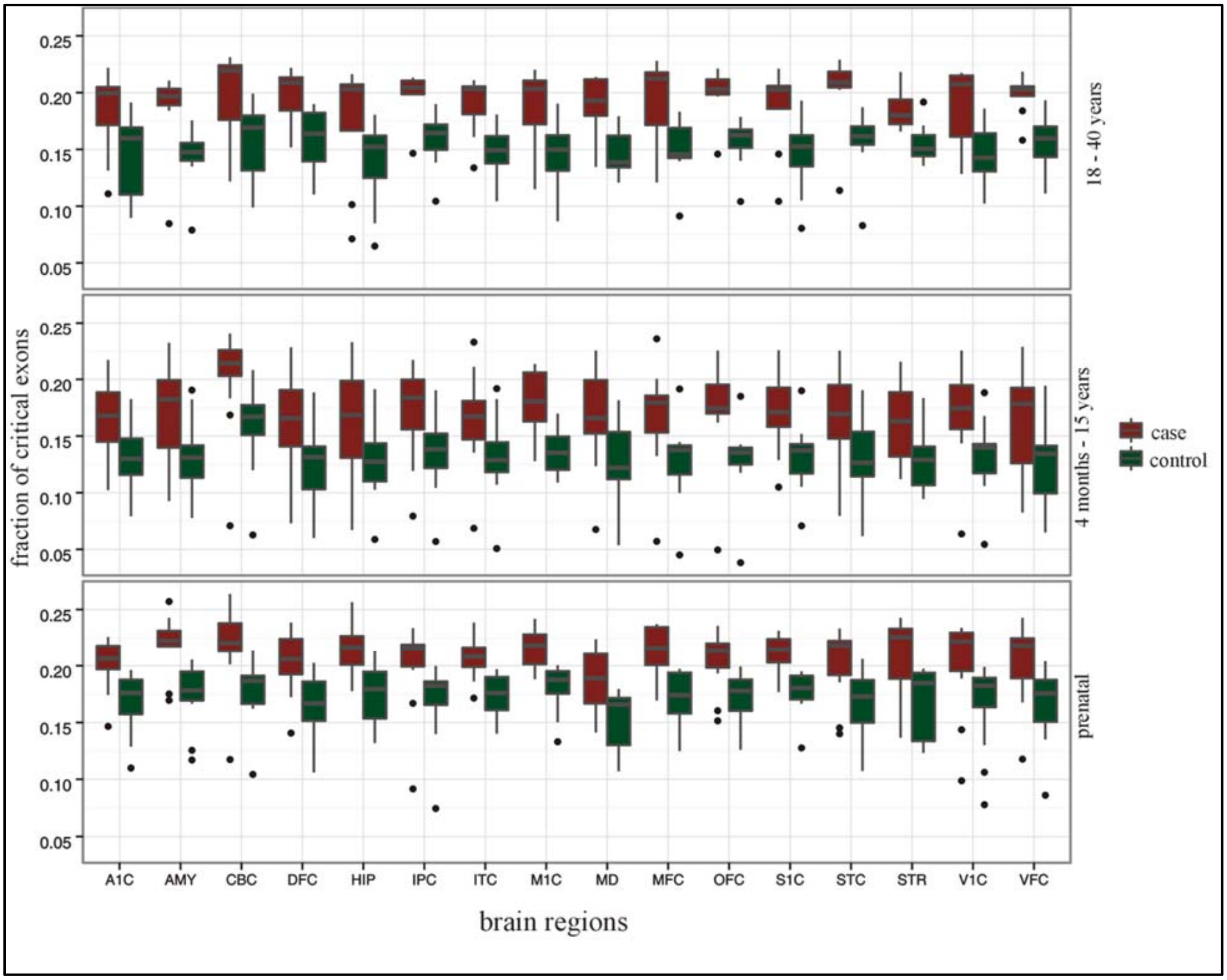

Figure S32 The proportion of critical exons impacted in the cases and the 873 platform matched population controls in 16 different brain regions. The p-values are shown in Figure 2.

Table S4 A summary whole-exome findings [see online Table S4.xlsx]

Table S5: The probability of truncating loss-of-function intolerance (pLI) for genes impacted by CNVs in Table 1.

case	ID	location	size (bp)	genes (pLI)
				de novo
Case A	200117	Xp22.33-Xq28	155,270,560	826 genes
Case B	234968	Xq13.1-Xq28	84,886,227	457 genes
		Xp22.33-Xq13.1	70,384,332	369 genes
Case C	212306	Xp22.31	1,686,421	*STS* (0.98) + *VCX* (0.58), *VCX3A* (0.51), *PNPLA4* (0.01), *HDHD1* (0.26)
		11q24.1	76,847	*SCN3B* (0.35), *GRAMD1B* (0.98)
		3q11.2	39,636	*EPHA6* (0.95)
Case D	208937	18p11.23, 18p11.31	1,146,375	*LAMA1* (0.0), *PTPRM* (1.0), *LRRC30* (0.0)
		Xp21.2	309,376	*DMD* (1.0), *FTHL17* (0.0)
Case E	216072	6q16.3	475,077	*GRIK2* (0.99)
Case F	216185	10p15.3	67,632	*DIP2C* (1.0)
Case G	222710	10p15.3	67,632	*DIP2C* (1.0)
		7q36.2	14,653	*DPP6* (0.97)
**Inherited: Decipher Syndromes /Cytogenetic chromosomal anomalies**				
Case G	222710	22q11.21	2,548,820	45 genes
Case H	216192	17p12	1,395,494	*PMP22* (0.83), *CDRT4* (0.24), *CDRT15* (0.01), *TEKT3* (0.0), *TVP23C-CDRT4* (0.0), *TVP23C* (0.0), *HS3ST3B1* (0.09), *COX10* (0.65), *CDRT1* (NA)
**Inherited: CNVs impacting neurodevelopmental or muscular function genes**				
Case I	219594	3p26.3	1,027,287	*CNTN6* (0.0), *CNTN4* (1.0), *CNTN4-AS2* (NA)
Case J	198195	8p23.1, 8p23.2	973,229	*MCPH1* (0.0), *XKR5* (NA), *DEFB1* (0.0), *DEFA5* (0.0), *DEFA6* (0.0), *AGPAT5* (0.01), *DEFA4* (0.27), *DEFA3* (0.49), *DEFA1* (NA), *ANGPT2* (0.45), *DEFA1B* (NA)
Case K	214281	5q11.2	504,651	*HSPB3* (0.0), *ARL15* (0.01)
Case L	216197	16p13.2	370,593	*ABAT* (0.37), *USP7* (1.0), *PMM2* (0.0), *CARHSP1* (0.0), *C16orf72* (0.99), *TMEM186* (0.0)
Case M	217737	8q24.3	255,856	*KCNK9* (0.13)
		7q36.3	251,267	*NCAPG2* (0.98), *ESYT2* (0.01)
Case N	247947	1q23.2	196,398	*KCNJ10* (0.60), *KCNJ9* (0.58), *IGSF8* (0.0), *IGSF9* (0.57), *PIGM* (0.0), *SLAMF9* (0.01), *TAGLN2* (0.0)
Case O	217932	3q26.31	176,370	*NAALADL2* (0.0)
Case P	208289	22q11.21	162,405	*DGCR2* (0.0), *DGCR14* (0.0), *TSSK2* (0.0), *GSC2* (0.04), *SLC25A1* (0.63), *CLTCL1* (0.0)
Case Q	221713	7q35	114,399	*CNTNAP2* (0.0)
Case R	208341	5p15.31	77,527	*SEMA5A* (0.0)
Case S	234267	9q33.1	40,814	*ASTN2* (0.99)
Case T	239838	22q12.3	34,122	*RFPL2* (0.0), *SLC5A4* (0.0)
Case U	208290	18p11.31	25,582	*DLGAP1* (0.99)
Case V	199743	16q24.3	24,064	*ZNF778* (0.0)
Case W	209038	17p11.2	16,602	*MYO15A* (NA)

Table S6 Genes impacted by CNVs in previous publications and the present study emphasizing CNVs shared between studies.

**sample**	**chr**	**start (hg19)**	**end (hs19)**	**type**	**genes***	**studies**
219594	chr3	1196554	2223840	del	*CNTN6*	Current project
Segel-10	chr3	1328461	1849338	del	*CNTN6*	Segel2015[52]
221713	chr7	146988081	147102479	del	*CNTNAP2*	Current project
Segel-19	chr7	147975925	148248042	dup	*CNTNAP2*	Segel2015
10-006C	chrX	31402175	32134404	dup	*DMD*	Oskoui2015[51]
216182	chrX	31873881	31894898	dup	*DMD*	Current project
208937	chrX	31074493	31383868	dup	*DMD*	Current project
10-022C	chrX	6697642	7041357	del	*HDHD1*	Oskoui2015
212306	chrX	6449237	8135657	del	*HDHD1*	Current project
198195	chr8	6025992	6999220	dup	*MCPH1*	Current project
P025	chr8	6084826	6304250	dup	*MCPH1*	McMichael2014[20]
216185	chr9	140370923	140418419	del	*PNPLA7*	Current project
12-0001C	chr9	140370972	140427037	del	*PNPLA7*	Oskoui2015
239838	chr7	84260948	84661969	dup	*SEMA3D*	Current project
13-031C	chr7	84267724	84660958	dup	*SEMA3D*	Oskoui2015
208351	chr5	101662158	101860664	dup	*SLCO6A1*	Current project
P053	chr5	101735294	101735429	del	*SLCO6A1*	McMichael2014

*only impacted genes are shows for each CNV.

Control sample permissions

We obtained the KORA, COGEND and Health ABC (HABC) control cohorts along with permission for use, from the database of Genotypes and Phenotypes (dbGap; https://www.ncbi.nlm.nih.gov/gap) through accession numbers phs000169.v1.p1 (Whole Genome Association Study of Visceral Adiposity in the HABC Study), phs000303.v1.p1 (Genetic Epidemiology of Refractive Error in the KORA Study) and phs000404.v1.p1 (COGEND; The Genetic Architecture of Smoking and Smoking Cessation). The Division of Aging Biology and the Division of Geriatrics and Clinical Gerontology, National Institute on Aging provided support for the "Center for Inherited Disease Research Visceral Adiposity Study". HABC Study Investigators provided study coordination and assistance with phenotype harmonization and genotype cleaning. The National Eye Institute provided support for the KORA data set that was obtained from the NEI Refractive Error Collaboration Database. Genotyping of the COGEND samples was conducted at the Center for Inherited Disease Research and support was provided by 1 X01 HG005274-01. The Gene Environment Association Studies (GENEVA) Coordinating Center (U01HG004446) provided study coordination and assistance with genotype cleaning for these samples. The COGEND (P01 CA089392) and the University of Wisconsin Transdisciplinary Tobacco Use Research Center (P50 DA019706 and P50 CA084724) provided the support for sample collection for the COGEND samples and data sets. The contents of this article are solely the responsibility of the authors and do not necessarily represent the official views of the NIH.

ACKNOWLEDGMENTS

The authors thank Jennifer Howe, John Wei, Sergio Pereira, Zhuozhi Wang, Wilson Sung, Gaganjot Kaur, Thomas Nalpathamkalam, Tara Paton, Giovanna Pellecchia, Ryan K.C. Yuen, Anath Lionel, Ada Chan, Marc Woodbury-Smith, Sylvia Lamoureux, Alireza Mowjoodi, Chao Lu, Ting Wang, Xiaolin Wang, Zhanqin Liu, Honglei Yu, Karen Ho, Zhizhou Hu, Kozue Samler, Lia D'Abate, and Janet Buchanan for technical assistance and helpful discussions. We would also like to thank Jackie Down, Shannon Geldart, Rhiannon Hicks, Lisa-Marie Langaigne, Nicole Murphy, Heather Ridgway, Jane Sandercock, Julie Wilson, and Melody Yuen for their assistance with data collection.

References:

1. Uddin M, Thiruvahindrapuram B, Walker S, et al. A high-resolution copy-number variation resource for clinical and population genetics. *Genet Med.* 2014.
2. Zarrei M, MacDonald JR, Merico D, Scherer SW. A copy number variation map of the human genome. *Nat Rev Genet.* 2015;16(3):172-183.
3. Pinto D, Delaby E, Merico D, et al. Convergence of genes and cellular pathways dysregulated in autism spectrum disorders. *Am J Hum Genet.* 2014;94(5):677-694.
4. Krawczak M, Nikolaus S, von Eberstein H, Croucher PJ, El Mokhtari NE, Schreiber S. PopGen: population-based recruitment of patients and controls for the analysis of complex genotype-phenotype relationships. *Community Genet.* 2006;9(1):55-61.
5. Stewart AF, Dandona S, Chen L, et al. Kinesin family member 6 variant Trp719Arg does not associate with angiographically defined coronary artery disease in the Ottawa Heart Genomics Study. *J Am Coll Cardiol.* 2009;53(16):1471-1472.
6. Verhoeven VJ, Hysi PG, Wojciechowski R, et al. Genome-wide meta-analyses of multiancestry cohorts identify multiple new susceptibility loci for refractive error and myopia. *Nat Genet.* 2013;45(3):314-318.
7. Bierut LJ, Madden PA, Breslau N, et al. Novel genes identified in a high-density genome wide association study for nicotine dependence. *Hum Mol Genet.* 2007;16(1):24-35.
8. Bierut LJ, Agrawal A, Bucholz KK, et al. A genome-wide association study of alcohol dependence. *Proc Natl Acad Sci U S A.* 2010;107(11):5082-5087.
9. Cotterchio M, Boucher BA, Manno M, Gallinger S, Okey AB, Harper PA. Red meat intake, doneness, polymorphisms in genes that encode carcinogen-metabolizing enzymes, and colorectal cancer risk. *Cancer Epidemiol Biomarkers Prev.* 2008;17(11):3098-3107.
10. Coviello AD, Haring R, Wellons M, et al. A genome-wide association meta-analysis of circulating sex hormone-binding globulin reveals multiple Loci implicated in sex steroid hormone regulation. *PLoS Genet.* 2012;8(7):e1002805.
11. Genomes Project C, Abecasis GR, Auton A, et al. An integrated map of genetic variation from 1,092 human genomes. *Nature.* 2012;491(7422):56-65.
12. Kang HJ, Kawasawa YI, Cheng F, et al. Spatio-temporal transcriptome of the human brain. *Nature.* 2011;478(7370):483-489.
13. Uddin M, Tammimies K, Pellecchia G, et al. Brain-expressed exons under purifying selection are enriched for de novo mutations in autism spectrum disorder. *Nat Genet.* 2014;46(7):742-747.
14. Uddin M, Pellecchia G, Thiruvahindrapuram B, et al. Indexing Effects of Copy Number Variation on Genes Involved in Developmental Delay. *Scientific Reports.* 2016;6:28663.
15. Warde-Farley D, Donaldson SL, Comes O, et al. The GeneMANIA prediction server: biological network integration for gene prioritization and predicting gene function. *Nucleic Acids Res.* 2010;38(Web Server issue):W214-220.
16. Pulvers JN, Journiac N, Arai Y, Nardelli J. MCPH1: a window into brain development and evolution. *Front Cell Neurosci.* 2015;9:92.
17. Trimborn M, Bell SM, Felix C, et al. Mutations in microcephalin cause aberrant regulation of chromosome condensation. *Am J Hum Genet.* 2004;75(2):261-266.
18. Ozgen HM, van Daalen E, Bolton PF, et al. Copy number changes of the microcephalin 1 gene (MCPH1) in patients with autism spectrum disorders. *Clin Genet.* 2009;76(4):348-356.

19. Perche O, Menuet A, Marcos M, et al. Combined deletion of two Condensin II system genes (NCAPG2 and MCPH1) in a case of severe microcephaly and mental deficiency. *Eur J Med Genet.* 2013;56(11):635-641.
20. McMichael G, Girirajan S, Moreno-De-Luca A, et al. Rare copy number variation in cerebral palsy. *Eur J Hum Genet.* 2014;22(1):40-45.
21. Kolb SJ, Snyder PJ, Poi EJ, et al. Mutant small heat shock protein B3 causes motor neuropathy: utility of a candidate gene approach. *Neurology.* 2010;74(6):502-506.
22. Sugiyama Y, Suzuki A, Kishikawa M, et al. Muscle develops a specific form of small heat shock protein complex composed of MKBP/HSPB2 and HSPB3 during myogenic differentiation. *J Biol Chem.* 2000;275(2):1095-1104.
23. Ehling P, Bittner S, Bobak N, et al. Two pore domain potassium channels in cerebral ischemia: a focus on K2P9.1 (TASK3, KCNK9). *Exp Transl Stroke Med.* 2010;2(1):14.
24. Heurteaux C, Bertaina V, Widmann C, Lazdunski M. K+ channel openers prevent global ischemia-induced expression of c-fos, c-jun, heat shock protein, and amyloid beta-protein precursor genes and neuronal death in rat hippocampus. *Proc Natl Acad Sci U S A.* 1993;90(20):9431-9435.
25. Lauritzen I, De Weille JR, Lazdunski M. The potassium channel opener (-)-cromakalim prevents glutamate-induced cell death in hippocampal neurons. *J Neurochem.* 1997;69(4):1570-1579.
26. Holter J, Carter D, Leresche N, Crunelli V, Vincent P. A TASK3 channel (KCNK9) mutation in a genetic model of absence epilepsy. *J Mol Neurosci.* 2005;25(1):37-51.
27. Barel O, Shalev SA, Ofir R, et al. Maternally inherited Birk Barel mental retardation dysmorphism syndrome caused by a mutation in the genomically imprinted potassium channel KCNK9. *Am J Hum Genet.* 2008;83(2):193-199.
28. Parolin Schnekenberg R, Perkins EM, Miller JW, et al. De novo point mutations in patients diagnosed with ataxic cerebral palsy. *Brain.* 2015.
29. Neusch C, Rozengurt N, Jacobs RE, Lester HA, Kofuji P. Kir4.1 potassium channel subunit is crucial for oligodendrocyte development and in vivo myelination. *J Neurosci.* 2001;21(15):5429-5438.
30. Pique LM, Brennan ML, Davidson CJ, Schaefer F, Greinwald J, Jr., Schrijver I. Mutation analysis of the SLC26A4, FOXI1 and KCNJ10 genes in individuals with congenital hearing loss. *PeerJ.* 2014;2:e384.
31. Yang T, Gurrola JG, 2nd, Wu H, et al. Mutations of KCNJ10 together with mutations of SLC26A4 cause digenic nonsyndromic hearing loss associated with enlarged vestibular aqueduct syndrome. *Am J Hum Genet.* 2009;84(5):651-657.
32. Reichold M, Zdebik AA, Lieberer E, et al. KCNJ10 gene mutations causing EAST syndrome (epilepsy, ataxia, sensorineural deafness, and tubulopathy) disrupt channel function. *Proc Natl Acad Sci U S A.* 2010;107(32):14490-14495.
33. Bockenhauer D, Feather S, Stanescu HC, et al. Epilepsy, ataxia, sensorineural deafness, tubulopathy, and KCNJ10 mutations. *N Engl J Med.* 2009;360(19):1960-1970.
34. Ciruela F, Fernandez-Duenas V, Sahlholm K, et al. Evidence for oligomerization between GABAB receptors and GIRK channels containing the GIRK1 and GIRK3 subunits. *Eur J Neurosci.* 2010;32(8):1265-1277.
35. Herman MA, Sidhu H, Stouffer DG, et al. GIRK3 gates activation of the mesolimbic dopaminergic pathway by ethanol. *Proc Natl Acad Sci U S A.* 2015;112(22):7091-7096.
36. Hansen M, Walmod PS. IGSF9 family proteins. *Neurochem Res.* 2013;38(6):1236-1251.

37. Mishra A, Traut MH, Becker L, Klopstock T, Stein V, Klein R. Genetic evidence for the adhesion protein IgSF9/Dasm1 to regulate inhibitory synapse development independent of its intracellular domain. *J Neurosci.* 2014;34(12):4187-4199.
38. Millson A, Lagrave D, Willis MJ, Rowe LR, Lyon E, South ST. Chromosomal loss of 3q26.3-3q26.32, involving a partial neuroligin 1 deletion, identified by genomic microarray in a child with microcephaly, seizure disorder, and severe intellectual disability. *Am J Med Genet A.* 2012;158A(1):159-165.
39. Shifman S, Levit A, Chen ML, et al. A complete genetic association scan of the 22q11 deletion region and functional evidence reveal an association between DGCR2 and schizophrenia. *Hum Genet.* 2006;120(2):160-170.
40. Wang H, Duan S, Du J, et al. Transmission disequilibrium test provides evidence of association between promoter polymorphisms in 22q11 gene DGCR14 and schizophrenia. *Journal of Neural Transmission.* 2006;113(10):1551-1561.
41. Mosca-Boidron AL, Gueneau L, Huguet G, et al. A de novo microdeletion of SEMA5A in a boy with autism spectrum disorder and intellectual disability. *Eur J Hum Genet.* 2016;24(6):838-843.
42. Sand PG. SEMA5A in Parkinson's disease. *Eur Rev Med Pharmacol Sci.* 2015;19(2):182-183.
43. Zhu B, Chen C, Xue G, et al. The SEMA5A gene is associated with hippocampal volume, and their interaction is associated with performance on Raven's Progressive Matrices. *Neuroimage.* 2013;88C:181-187.
44. Lionel AC, Tammimies K, Vaags AK, et al. Disruption of the ASTN2/TRIM32 locus at 9q33.1 is a risk factor in males for autism spectrum disorders, ADHD and other neurodevelopmental phenotypes. *Hum Mol Genet.* 2014;23(10):2752-2768.
45. Wilson PM, Fryer RH, Fang Y, Hatten ME. Astn2, a novel member of the astrotactin gene family, regulates the trafficking of ASTN1 during glial-guided neuronal migration. *J Neurosci.* 2010;30(25):8529-8540.
46. Bonnefont J, Nikolaev SI, Perrier AL, et al. Evolutionary forces shape the human RFPL1,2,3 genes toward a role in neocortex development. *Am J Hum Genet.* 2008;83(2):208-218.
47. Gazzellone MJ, Zarrei M, Burton CL, et al. Uncovering obsessive-compulsive disorder risk genes in a pediatric cohort by high-resolution analysis of copy number variation. *J Neurodev Disord.* 2016;8:36.
48. Willemsen MH, Fernandez BA, Bacino CA, et al. Identification of ANKRD11 and ZNF778 as candidate genes for autism and variable cognitive impairment in the novel 16q24.3 microdeletion syndrome. *Eur J Hum Genet.* 2010;18(4):429-435.
49. Sacharow S, Li D, Fan YS, Tekin M. Familial 16q24.3 microdeletion involving ANKRD11 causes a KBG-like syndrome. *Am J Med Genet A.* 2012;158A(3):547-552.
50. Miyagawa M, Nishio SY, Hattori M, et al. Mutations in the MYO15A gene are a significant cause of nonsyndromic hearing loss: massively parallel DNA sequencing-based analysis. *Ann Otol Rhinol Laryngol.* 2015;124 Suppl 1:158S-168S.
51. Oskoui M, Gazzellone MJ, Thiruvahindrapuram B, et al. Clinically relevant copy number variations detected in cerebral palsy. *Nat Commun.* 2015;6:7949.
52. Segel R, Ben-Pazi H, Zeligson S, et al. Copy number variations in cryptogenic cerebral palsy. *Neurology.* 2015;84(16):1660-1668.
